# TPX2/Aurora kinase A signaling as a potential therapeutic target in genomically unstable cancer cells

**DOI:** 10.1038/s41388-018-0470-2

**Published:** 2018-09-03

**Authors:** Stephanie E. van Gijn, Elles Wierenga, Nathalie van den Tempel, Yannick P. Kok, Anne Margriet Heijink, Diana C. J. Spierings, Floris Foijer, Marcel A. T. M. van Vugt, Rudolf S. N. Fehrmann

**Affiliations:** 1 Department of Medical Oncology, University Medical Center Groningen, University of Groningen, Groningen, The Netherlands; 2 European Research Institute for the Biology of Ageing, University Medical Center Groningen, University of Groningen, Groningen, The Netherlands

**Keywords:** Targeted therapies, Homologous recombination

## Abstract

Genomic instability is a hallmark feature of cancer cells, and can be caused by defective DNA repair, for instance due to inactivation of *BRCA2*. Paradoxically, loss of *Brca2* in mice results in embryonic lethality, whereas cancer cells can tolerate *BRCA2* loss. This holds true for multiple DNA repair genes, and suggests that cancer cells are molecularly “rewired” to cope with defective DNA repair and the resulting high levels of genomic instability. In this study, we aim to identify genes that genomically unstable cancer cells rely on for their survival. Using functional genomic mRNA (FGmRNA) profiling, 16,172 cancer samples were previously ranked based on their degree of genomic instability. We analyzed the top 250 genes that showed a positive correlation between FGmRNA levels and the degree of genomic instability, in a co-functionality network. Within this co-functionality network, a strong cluster of 11 cell cycle-related genes was identified, including TPX2. We then assessed the dependency on these 11 genes in the context of survival of genomically unstable cancer cells, induced by *BRCA2* inactivation. Depletion of TPX2 or its associated kinase Aurora-A preferentially reduced cell viability in a panel of BRCA2-deficient cancer cells. In line with these findings, BRCA2-depleted and *BRCA2*-mutant human cell lines, or tumor cell lines derived from *Brca2*^–/–^;*p53*^–/–^ mice showed increased sensitivity to the Aurora-A kinase inhibitor alisertib, with delayed mitotic progression and frequent mitotic failure. Our findings reveal that BRCA2-deficient cancer cells show enhanced sensitivity to inactivation of TPX2 or its partner Aurora-A, which points at an actionable dependency of genomically unstable cancers.

## Introduction

Genomic instability is a common feature of human cancers, and drives the progressive accumulation of genomic aberrations, including somatic copy-number alterations (SCNAs) and segmental or whole-chromosome aneuploidies [1]. The extent of genomic instability varies across different tumors, ranging from tumors with relatively few SCNAs (e.g., acute myeloid leukemia) to tumors that harbor excessive SCNAs (e.g., high-grade serous ovarian cancer (HGSOC) and triple-negative breast cancer (TNBC)) [2–4]. Tumors with relatively high levels of genomic instability typically behave aggressively, with early (visceral) metastatic spread and have a poor prognosis. Unfortunately, these tumors lack the “oncogenic drivers” that are currently actionable, omitting these patients to benefit from the available molecular targeted agents [5, 6].

Genome maintenance is tightly controlled by checkpoint mechanisms that coordinate cell cycle progression with DNA repair, and coordinate faithful chromosome segregation during mitosis [7]. Genomic instability can be caused, among other events, by mutations in DNA repair genes. For instance, tumors arising in women carrying germline heterozygous mutations in the homologous recombination (HR) DNA repair genes *BRCA1* or *BRCA2* are extensively genomically unstable [8].

During the S/G_2_-phase of the cell cycle, HR repair is required to faithfully repair DNA double-stranded breaks (DSBs) using a sister chromatid as the repair template [9]. Both BRCA1 and BRCA2 act to facilitate the loading of RAD51 recombinase, which is ultimately responsible for strand invasion and recombination [10]. When HR is defective, error-prone DNA repair pathways, including nonhomologous end joining (NHEJ) and single-strand annealing (SSA) are utilized, resulting in loss of genomic integrity [11].

The requirement of HR for cellular viability is illustrated by the phenotype observed in *Brca2* knockout mouse models, as *Brca*2-deficient mice die early in embryogenesis, with elevated levels of DNA damage that lead to cell cycle arrest [12, 13]. In stark contrast, tumor cells are apparently able to cope without *BRCA2*. These observations are not unique to BRCA2 loss, as cellular viability also reduces upon loss of the HR repair factor BRCA1 [12, 14]. In part, survival of HR-defective cancer cells can be explained by loss of the tumor-suppressor p53. In line with this notion, *BRCA1* and *BRCA2*-mutant cancer cells almost invariably have *TP53* mutations; however, the combined inactivation of *BRCA2* or *BRCA1* and *TP53* still yields cells that display impaired proliferation [12]. Very likely, multiple other genetic alterations influence the viability of HR-defective cancer cells.

Unraveling how genomically unstable tumors are molecularly “rewired” to survive high levels of genomic instability may provide a strategy to target these tumors. Previous studies have shown that genomically unstable tumors can show an addiction toward genes that secure their survival [15, 16]. Targeting these genes could result in the development of molecular treatment regimens tailored to patients with genomically unstable cancers.

Previously, we employed functional genomic mRNA (FGmRNA) profiling to determine the degree of genomic instability in 16,172 patient-derived tumor samples [2]. Herein, associations between the expression of individual genes and their association to the degree of genomic instability were assessed. In the current study, we found that the top 250 genes positively associated with the degree of genomic instability, revealed a strong network of genes with shared functionality implicated in the cell cycle, including TPX2. The relevance of the genes within this cluster was tested using in vitro models, in which genomic instability was induced by *BRCA2* inactivation. Our findings show that BRCA2-deficient cancer cells show enhanced sensitivity to inactivation of TPX2 or its partner Aurora-A. These results point at actionable dependencies of genomically unstable cancers on faithful mitotic processes.

## Results

### Identification of a cluster of genes of which individual gene expression positively associates to the degree of genomic instability

**Fig. 1:**
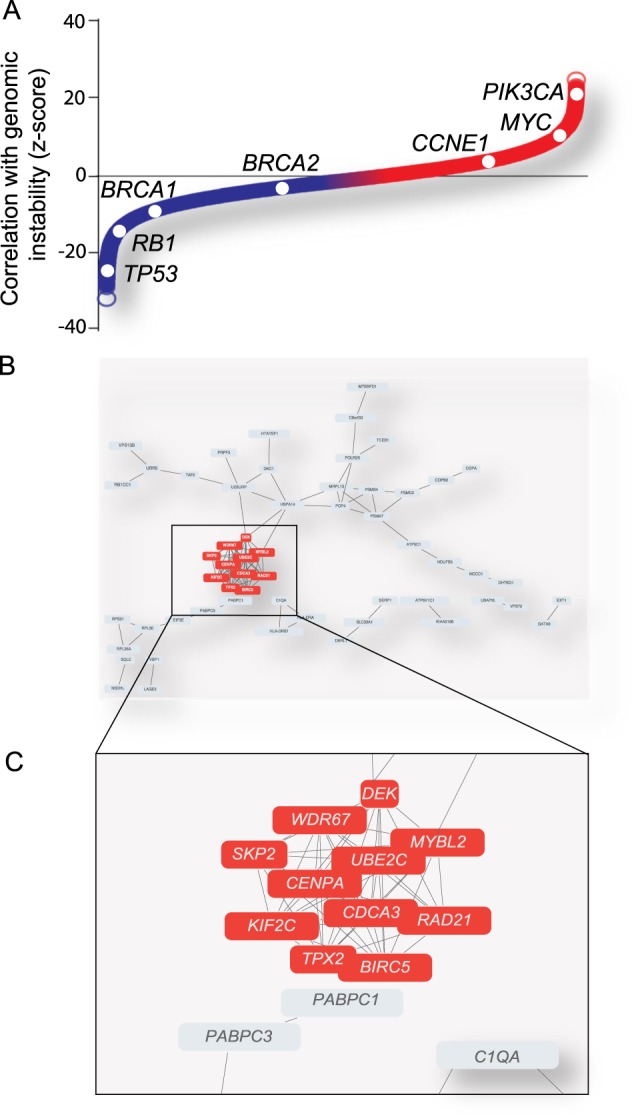
Identification of a cluster of genes of which individual gene expression associates to the degree of genomic instability. **a** Ranked associations of mRNA expression of individual genes and their association to the degree of genomic instability (*z*-scores). **b**, **c** Co-functionality analysis based on similar biological processes of the top 250 genes (**b**), of which expression is positively associated with genomic instability, revealed a cluster of 11 genes (correlation coefficient > 0.5) (**c**)

To identify genes that are potentially involved in the molecular “rewiring” of tumor cells to cope with high levels of genomic instability, we built on a previously described data set, in which a transcriptome-wide association analysis was performed between the expression of individual genes and the degree of genomic instability in 16,172 tumor samples [2] (Fig. 1a). We selected the top 250 genes that showed the strongest association between elevated mRNA levels and the degree of genomic instability (Fig. 1b). These 250 genes were analyzed for predicted co-functionality, which revealed a strong cluster of 11 genes (with a correlation coefficient >0.5), implicated in cell cycle regulation, including *TPX2* (Fig. 1c).

### TPX2 depletion preferentially affects viability in BRCA2-depleted cells

**Fig. 2:**
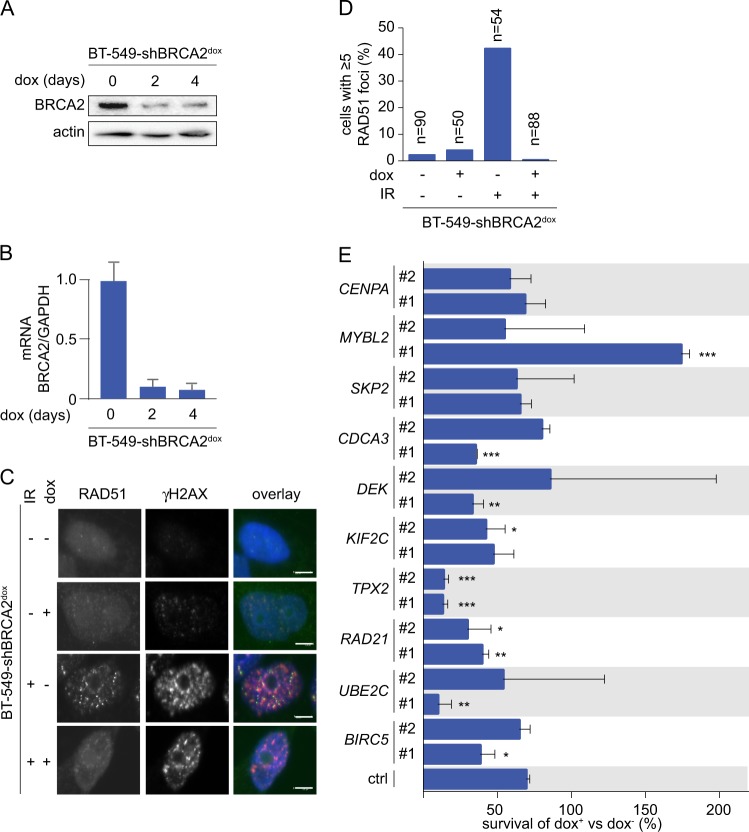
TPX2 depletion preferentially affects cell viability in BRCA2-deficient cancer cells. **a** BT-549-shBRCA2^dox^ cells were left untreated or were treated with doxycycline (2 or 4 days), and subsequently harvested for western blotting for BRCA2 and actin. **b** BT-549-shBRCA2^dox^ cells were treated as in panel A, and mRNA expression levels of BRCA2 were analyzed relative to GAPDH using qRT-PCR. **c** BT-549-shBRCA2^dox^ cells were grown on coverslips and treated with doxycycline (3 days) and/or irradiated (IR, 5 Gy) as indicated. At 3 h after irradiation, cells were fixed and analyzed for RAD51 and γH2AX foci formation. Scale bars represent 5 μm. **d** Percentages of cells with ≥5 RAD51 foci per nucleus are indicated. (*n* ≥ 50 per condition). **e** BT-549-shBRCA2^dox^ cells were treated with doxycycline (3 days) and were subsequently transfected with indicated siRNAs. A total of 30,000 cells were plated 48 h following transfection. Viable cells were counted 5 days later. Percentages of cell survival of doxycycline-treated vs untreated cells are depicted. Error bars indicate standard deviations of two experimental replicates. Unpaired two-tailed *t* tests were used to test for statistical significance (*p ≤ 0.05, **p ≤ 0.01, ***p ≤ 0.001)

To test the relevance of the identified 11 genes in cellular survival of genomically unstable cancer cells, we modeled genomic instability in vitro by doxycycline-inducible shRNA-mediated depletion of the DNA repair protein BRCA2. Treatment of BT-549-shBRCA2^dox^ cells with doxycycline resulted in a robust reduction of BRCA2 protein (Fig. 2a) and *BRCA2* mRNA (Fig. 2b). More importantly, a functional assay to test the ability to repair through HR by analysis of irradiation-induced RAD51 foci, showed that doxycycline-induced depletion of BRCA2 resulted in a virtual complete loss of RAD51 irradiation-induced foci (IRIF) formation to DSBs marked by γH2AX foci (42% vs 0.4% of cells with ≥5 RAD51 foci per cell, in control-depleted vs BRCA2-depleted cells) (Fig. 2c, d).

siRNA was used to deplete each of the selected 11 genes in BT-549-shBRCA2^dox^ cells. Despite using five independent siRNA sequences, we were not able to deplete WDR67, and for this reason left this gene out for further analysis. Doxycycline treatment prior to transfection did not affect siRNA efficiency, as no significant differences in reduction of mRNA expression of the ten genes in the presence or absence of doxycycline were observed by qRT-PCR (Suppl. Figure 1a), nor did doxycycline treatment affect cell cycle progression (Suppl. Figure 1b). Cell survival was assessed at 5 days after siRNA transfection, using two independent siRNAs per gene, in the absence or presence of doxycycline (Fig. 2e, Suppl. Figure 1c). *BIRC5*, *UBE2C*, and *RAD21* appeared essential in our setup, as depletion of these genes led to low cell counts, both in BRCA2-deficient and BRCA2-proficient cells. Depletion of *CDCA3*, *SKP2*, *MYBL2*, or *CENPA* did not affect survival regardless of BRCA2 status (Fig. 2e, Suppl. Figure 1c). In contrast, *TPX2*, *KIF2C*, and *DEK* were conditionally required, as depletion of these genes led to significantly lower numbers of viable cells in BRCA2-deficient cells compared to BRCA2-proficient cells (Fig. 2e, Suppl. Figure 1c). Depletion of TPX2, a microtubule-associated protein, led to the largest differential levels of viable cells when comparing BRCA2-deficient with BRCA2-proficient BT-549 cells (siTPX2 #1 *p* = 0.0002; siTPX2, #2 *p* = 0.0002) (Fig. 2e, Suppl. Figure 1c).

### TPX2/Aurora-A depletion affects viability in BRCA2-depleted breast cancer cells

**Fig. 3:**
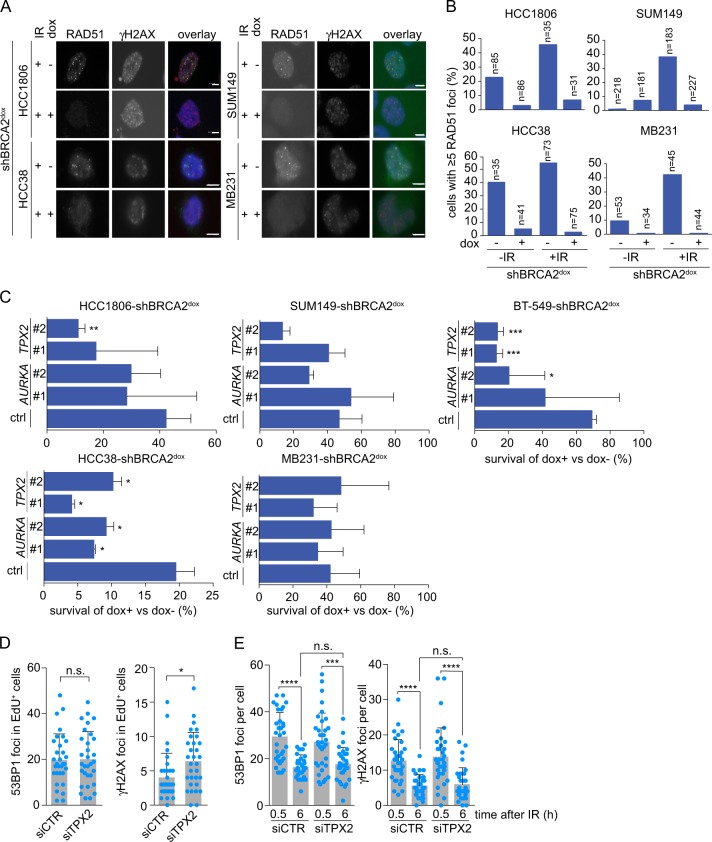
Depletion of TPX2 or Aurora-A reduces cell viability of BRCA2-deficient breast cancer cells. **a** HCC1806-shBRCA2^dox^, HCC38-shBRCA2^dox^, SUM149-shBRCA2^dox^, and MB231-shBRCA2^dox^ were grown on coverslips and treated with doxycycline (3 days) and/or irradiated (IR, 5 Gy) as indicated. Subsequently, cells were stained for RAD51 and γH2AX. Scale bars represent 5 μm. **b** Quantification of results from **a**. Percentages of cells with ≥5 RAD51 foci per nucleus are indicated (*n* ≥ 31). **c** Percentages of cell survival of doxycycline-treated cells vs untreated cells, transfected with indicated siRNAs. Unpaired two-tailed *t* tests were used to test for statistical significance (*p ≤ 0.05, **p ≤ 0.01, ***p ≤ 0.001). **d** BT-549 cells were transfected with siTPX2 or control siRNA (CTR). Cells were grown on coverslips for 3 days after which they were incubated with EdU conjugated to azide-Alexa 488 (10 μM) for 15 min. Subsequently, cells were fixed and stained for 53BP1 and γH2AX. Amounts of 53BP1 and γH2AX foci per cell of at least 30 EdU-positive cells were counted. Means and standard deviations are depicted. Mann–Whitney *U* tests were used to analyze statistical significance (*p ≤ 0.05, ** = *p* ≤ 0.01, ***p ≤ 0.001, ns not significant). **e** BT-549 cells were transfected as in **d**, irradiated (IR, 5 Gy), and fixated 0.5 or 6 h after irradiation. Amounts of 53BP1 and γH2AX foci per cell were counted. Means and standard deviations are depicted. Mann–Whitney *U* tests were used to analyze statistical significance (*p ≤ 0.05, **p ≤ 0.01, ***p ≤ 0.001, ns = not significant)

To test whether the reduction in cell viability of BRCA2-deficient cells upon TPX2 depletion was also observed in other cancer models, we engineered four other breast cancer cell lines with doxycycline-inducible BRCA2 shRNAs: SUM149-shBRCA2^dox^, MDA-MB231-shBRCA2^dox^, HCC38-shBRCA2^dox^, and HCC1806-shBRCA2^dox^. Treatment with doxycycline reduced BRCA2 protein levels in all cell lines (Suppl. Figure 2), and resulted in impaired HR, as illustrated by the reduced amount of RAD51 IRIF formation (Fig. 3a, b). A robust reduction in cell viability of BRCA2-deficient cells compared to BRCA2-proficient cells after TPX2 depletion was observed in HCC38-shBRCA2^dox^ (siTPX#1, *p* = 0.01, siTPX2#2, *p* = 0.04) and HCC1806-shBRCA2^dox^ (siTPX2#2, *p* = 0.003) (Fig. 3c, Suppl. Figure 2b).

TPX2 is a microtubule-associated protein and a cofactor of Aurora-A kinase. Binding of TPX2 to Aurora-A leads to activation of and subsequent localization of Aurora-A to microtubules during mitosis to facilitate bipolar spindle formation and thus faithful chromosome segregation [17]. We next tested if Aurora-A inactivation using RNAi resulted in similar effects in BRCA2-depleted cells (Fig. 3c, Suppl. Figure 2b). Depletion of Aurora-A led to a reduction in cell survival of BRCA2-deficient cells compared to BRCA2-proficient cells in BT-549-shBRCA2^dox^ (siAURKA#2, *p* = 0.02) and HCC38-shBRCA2^dox^ cells (siAURKA#1, *p* = 0.02; siAURKA#2, *p* = 0.03) (Fig. 3c, Suppl. Figure 2b).

BRCA2-deficient cells have impaired DNA repair capacity, and may rely for their survival on residual DNA repair pathways, such as non-homologous end joining (NHEJ). To test if TPX2 depletion impacts on DNA repair capacity, we analyzed the amounts of DNA lesions in TPX2-depleted proliferating cells. Numbers of spontaneous 53BP1 foci were not increased, whereas a minor, but statistically significant increase in γ-H2AX foci was observed in EdU-positive cells (Fig. 3d). In response to IR, TPX2-depleted cells did not show delayed clearance of γ-H2AX and 53BP1 foci (Fig. 3e, Suppl. Figure 3f), suggesting that the observed effects of TPX2 depletion in BRCA2-depleted cells are not caused through interference with residual DNA repair capacity in BRCA2-depleted cells.

### Upregulation and downregulation of TPX2 levels affects mitotic fidelity

**Fig. 4:**
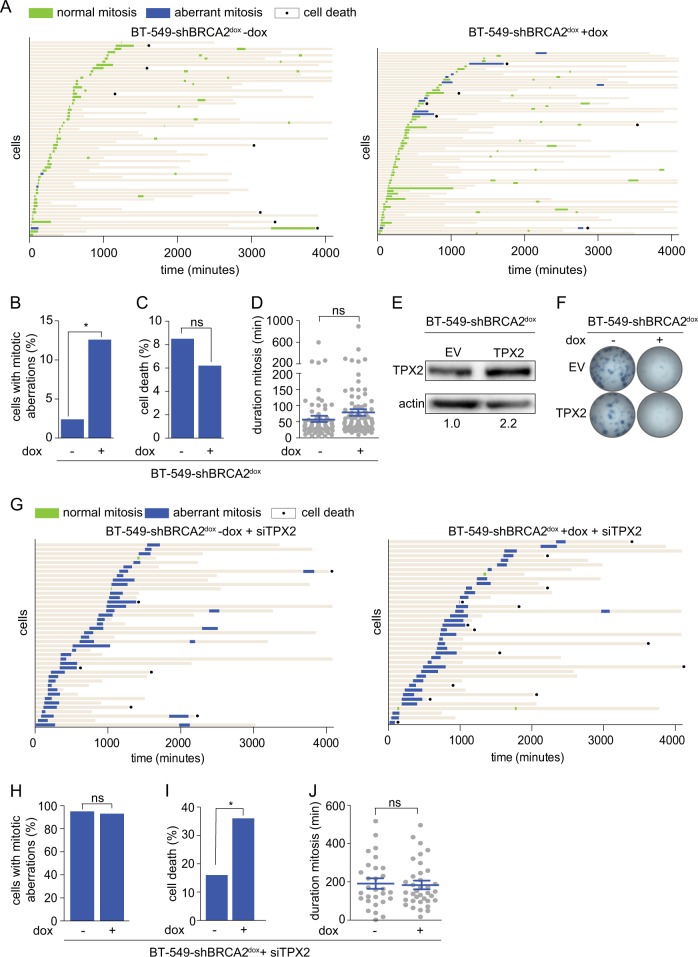
Depletion of TPX2 leads to aberrant mitoses. **a** BT-549-shBRCA2^dox^ cells, stably expressing H2B–GFP were treated with doxycycline (24 h), and subsequently followed with live-cell microscopy for 65 h. The left panel represents untreated cells, the right panel represents doxycycline-treated cells. Each bar represents a single cell: green bars indicate normal mitoses, blue bars indicate cells with aberrant mitoses, and black dots indicate cell death. **b** Percentages of BT-549-shBRCA2^dox^ cells, left untreated or treated with doxycycline, that showed aberrant mitoses (unpaired two-tailed *t* test, *p* = 0.03). **c** Percentages of BT-549-shBRCA2^dox^ cells, left untreated or treated with doxycycline, that died (unpaired two-tailed *t* test, *p* = 0.56). **d** Duration of mitosis in BT-549-shBRCA2^dox^ cells, treated with or without doxycycline. Means and standard errors of the mean are depicted (unpaired two-tailed *t* test, *p* = 0.12). **e** BT-549-shBRCA2^dox^ cells were infected with pBabe-EV or pBabe-TPX2, and immunoblotted for TPX2 and actin. **f** Clonogenic survival assay of BT-549-shBRCA2^dox^ cells, infected with pBabe-EV or pBabe-TPX2, and treated with or without doxycycline as indicated. **g** BT-549-shBRCA2^dox^ cells were transfected with TPX2 siRNA and treated with doxycycline or left untreated, and were followed with live-cell microscopy for 65 h. Each bar represents a single cell: green bars indicate normal mitoses, blue bars indicate cells with aberrant mitoses, and black dots indicate cell death. **h** Percentages of mitotic aberrations in BT-549-shBRCA2^dox^ cells transfected with TPX2 siRNA and treated with or without doxycycline (unpaired two-tailed *t* test, *p* = 0.58). **i** Percentages of cell death after mitosis in BT-549-shBRCA2^dox^ cells, transfected with TPX2 siRNA and treated with or without doxycycline (unpaired two-tailed t test, *p* = 0.02). **j** Duration of mitosis in BT-549-shBRCA2^dox^ cells transfected with TPX2 siRNA, and treated with or without doxycycline. Means and standard errors of the mean are depicted (unpaired two-tailed *t* test, ns not significant)

Since TPX2 depletion preferentially affects cell survival in BRCA2-depleted cancer cells and because TPX2 functions in mitotic spindle assembly, we examined whether progression through mitosis is aberrant in BRCA2-deficient cells. To this end, we stably transduced BT-549-shBRCA2^dox^ cells with GFP-tagged histone—H2B and assessed chromosome segregation and duration of mitosis using time-lapse microscopy (Fig. 4a). BRCA2-deficient cells displayed more aberrant mitoses (12.6% vs 2.4% in BRCA2-proficient cells, *p* = 0.03) (Fig. 4b). The number of cells undergoing cell death in the course of the experiment did not differ between BRCA2-proficient and BRCA2-deficient cells (8.5 vs 6.2% of cells that underwent mitosis, respectively, *p* = 0.56) (Fig. 4c). The mean duration of mitosis amounted to 57 min in BRCA2-proficient compared to 80 min in BRCA2-deficient cells (*p* = 0.12) (Fig. 4d).

Using FGmRNA profiling, we found that elevated mRNA levels of TPX2 were associated with a high degree of genomic instability. We argued that TPX2 amplification alone was likely not sufficient to rescue the loss of viability induced by BRCA2 inactivation since genomically unstable tumors typically harbor multiple structural chromosomal aberrations. Indeed, stably increased TPX2 expression upon retroviral transduction (leading to an ~2-fold increase in expression in BT-549-shBRCA2^dox^ cells), did not lead to a rescue of the loss of viability induced by BRCA2 depletion (Fig. 4e, f, and Suppl. Figure 3a, b). Also, TPX2 overexpression did not result in significantly different levels of Caspase-3 cleavage, a measure of apoptosis (Suppl. Figure 3c), nor was HR function influenced by TPX2 overexpression, as assessed by RAD51 IRIF formation (Suppl. Figure 3d, e). Rather, time-lapse microscopy revealed that overexpression of TPX2 significantly prolonged the duration of mitosis, and increased cell death and the amount of aberrant mitoses (Suppl. Figure 4a–d).

In an attempt to explain the reduction in cell survival, observed preferentially in BRCA2-deficient cells after TPX2 depletion, we assessed whether BRCA2-deficient cells might become more dependent on TPX2 for faithful completion of mitosis. For this purpose, we followed BT-549-shBRCA2^dox^ cells depleted of TPX2 using time-lapse microscopy (Fig. 4g). Depletion of TPX2 increased mitotic duration in both BRCA2-proficient and BRCA2-deficient cells (*p* = 0.78) (Fig. 4d, j), and the amount of mitotic aberrations increased in both BRCA2-proficient and BRCA2-deficient cells (*p* = 0.58) (Fig. 4b, h). Most of the mitotic aberrations involved failure to perform cytokinesis (data not shown). Depletion of TPX2 resulted in increased amounts of mitotic cells that eventually died (Fig. 4c). A robust increase in cell death was observed in BRCA2-deficient cells depleted of TPX2 compared to untreated BRCA2-deficient cells (Fig. 4c, i; *p* < 0.0001). Only a very subtle increase in cell death was observed in BRCA2-proficient cells depleted of TPX2 when compared to untreated BRCA2-proficient cells (Fig. 4c, i; *p* = 0.58). In line with the results of cell survival assays (Figs. 2e and 3c), failed mitoses in cells depleted of both TPX2 and BRCA2 resulted more frequently in cell death, when compared to BRCA2-proficient cells depleted of TPX2 (*p* = 0.02) (Fig. 4i).

### BRCA2-mutant cancer cells are more sensitive to Aurora-A inhibition

**Fig. 5:**
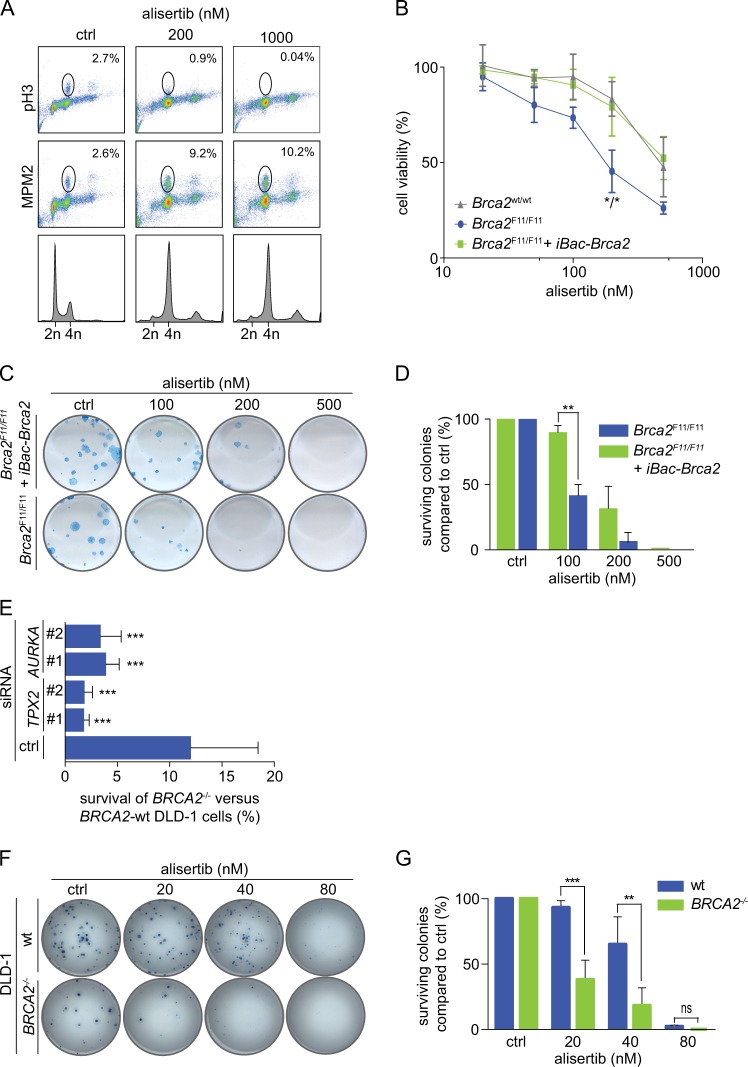
BRCA2-mutant cancer cells are differentially sensitive to Aurora-A inhibition. **a** BT-549 cells were left untreated or treated with 200 and 1000 nM of alisertib for 24 h. Cells were fixed and co-stained for pHH3, MPM2, and DNA was stained using propidium iodide. Cells were analyzed by flow cytometry. Percentages of cells stained positive for pHH3 and MPM2 are quantified. **b** Mouse mammary tumor cells were treated with indicated concentrations of alisertib. Results of three replicates were analyzed using ANOVA with Bonferroni post test, *p* < 0.05 at 200 nM. **c** Mouse mammary tumor cells were treated continuously with alisertib, and clonogenic cell survival was assessed. **d** Quantifications of colony numbers of three independent experiments as performed in **c**. Statistical analysis was done using ANOVA with Bonferroni post test, *p* < 0.01 at 100 nM. **e** Percentages of cell survival of *BRCA2*^*–/–*^ DLD-1 vs wt DLD-1 cells after transfection with siRNAs (unpaired two-tailed *t* test, TPX2: #1 *p* = 0.0006, #2 *p* = 0.0007, AURKA: #1 *p* = 0.0034, and for #2 *p* = 0.0026). **f** wt and *BRCA2*^*–/–*^ DLD-1 cells were treated with indicated concentrations of alisertib and clonogenic survival was assessed. **g** Quantifications of colony numbers of three independent experiments as performed in **g**. Statistical analysis was done using ANOVA with Bonferroni post test, *p* < 0.001 at 20 nM and, *p* < 0.01 at 40 nM

Since Aurora kinase A, which is associated with TPX2, is currently tested as a therapeutic target in cancer treatment [18], we investigated whether *BRCA2*-mutant cancer cells were more sensitive to chemical inhibition of Aurora-A. For this purpose, we tested the effects of the Aurora-A inhibitor alisertib in mouse mammary tumor cell lines derived from *Tp53*^–/–^;*Brca2*^wt/wt^ or *Tp53*^–/–^;*Brca2*^F11/F11^ mice (denoted as *Brca2*^wt/wt^, *Brca2*^F11/F11^). As a control, we used *Tp53*^–/–^;*Brca2*^F11/F11^ cells, reconstituted with a human BRCA2 cDNA (denoted *Brca2*^F11/F11^ + iBac-*Brca2*). As expected, RAD51 IRIF formation was impaired in the *Brca2*^F11/F11^ mouse mammary tumor cells, but was restored in *Brca2*^F11/F11^ + iBac-*Brca2* (Suppl. Figure 5a, b). Treatment with alisertib efficiently reduced phosphorylation of histone H3 (pH3) at serine-10, a substrate of Aurora-A, illustrating efficient target engagement at indicated doses (Fig. 5a). Simultaneously, alisertib treatment resulted in an accumulation of cells in G_2_/M-phase of the cell cycle (from 25.0% in control cells to 60.7% in cells treated with 200 nM alisertib) (Fig. 5a). Treatment with alisertib also increased the number of cells in mitosis (2.6% in untreated cells to 9.2% after treatment with 200 nM alisertib), as judged by staining for MPM2, a mitotic marker (Fig. 5a). Importantly, *Brca2*^F11/F11^ cells were significantly more sensitive to alisertib compared to *Brca2*^wt/wt^ or *Brca2*^F11/F11^ + iBac-*Brca*2 cells in short-term MTT assays (Fig. 5b) and clonogenic survival assays (Fig. 5c, d). Importantly, at the doses used, alisertib preferentially affected Aurora-A kinase activity, whereas at higher concentrations also the activity of Aurora-B and Aurora-C was inhibited (Suppl. Figure 6b). Also, while alisertib treatment preferentially affected BRCA2-depleted cells, the Aurora-B inhibitor ZM447439 affected cells regardless of BRCA2 status (Suppl. Figure 6c).

To validate whether reduced cell viability upon TPX2 and Aurora-A depletion is also observed in human *BRCA2*-mutant cells, we used human colorectal DLD-1 cells. *BRCA2*^–/–^ DLD-1 cells are HR-defective, as assessed by RAD51 IRIF formation (Suppl. Figure 5c, d). TPX2 and Aurora-A were successfully depleted in wt DLD-1 cells using siRNA (Suppl. Figure 5f), and the number of viable cells was assessed after 5 days (Fig. 5e and Suppl. Figure 5e). We found that in *BRCA2-*mutant cells, depletion of TPX2 and Aurora-A also reduced cell viability in preferentially *BRCA2*^–/–^ DLD-1 cells compared to *BRCA2* wt DLD-1 cells (siTPX2#1, *p* < 0.0001; siTPX2#2, *p* < 0.0001; siAURKA#1, *p* = 0.0034; and siAURKA#2, *p* = 0.0026, Fig. 5e). In line with the results of short-term survival assays, treatment with alisertib resulted in a preferential loss of viability in *BRCA2*^–/–^ DLD-1 cells when compared to *BRCA2* wt cells in clonogenic survival assays (Fig. 5f, g). Altogether, our data indicate that BRCA2 inactivation renders cancer cells dependent on the TPX2/Aurora-A signaling axis for their survival.

### Alisertib treatment impedes cytokinesis which is preferentially cytotoxic in BRCA2-deficient cells

**Fig. 6:**
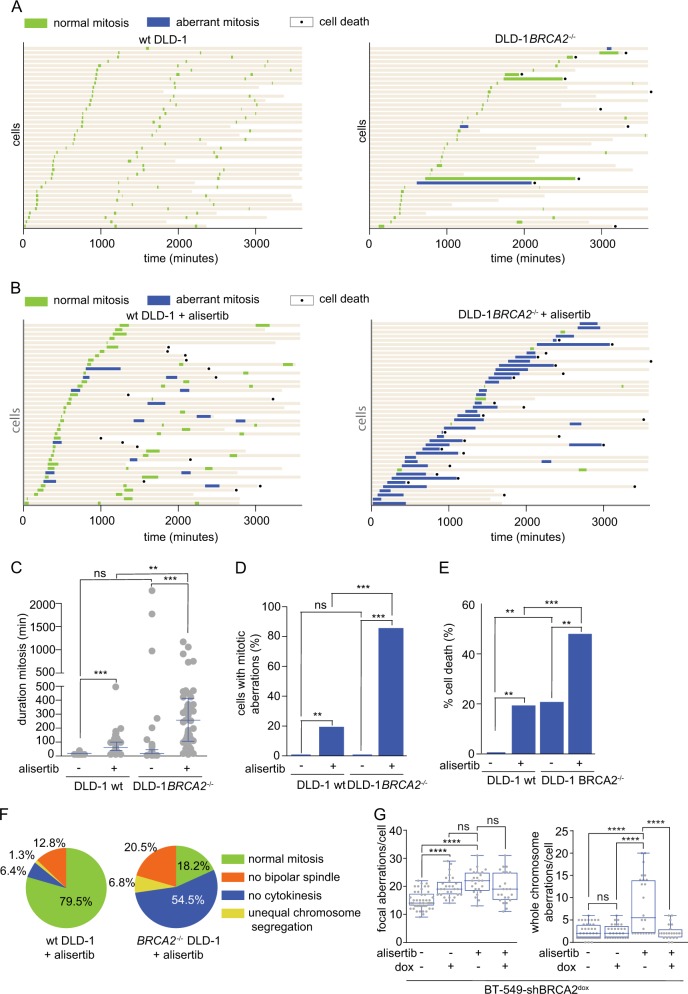
Depletion of Aurora-A impedes cytokinesis and is preferentially cytotoxic in BRCA2-deficient cells. **a** wt and *BRCA2*^*–/–*^ DLD-1 cells stably expressing H2B–GFP were followed with live-cell microscopy for 65 h. The left panel shows the mitotic behavior of wt DLD-1 cells and the right panel the mitotic behavior of *BRCA2*^*–/–*^ DLD-1 cells. Each bar represents a single cell: green bars indicate normal mitoses, blue bars indicate aberrant mitoses, and cell death is indicated with a black dot. **b** wt and *BRCA2*^*–/–*^ DLD-1 cells stably expressing H2B–GFP and treated with 200 nM alisertib. Graph depictions are similar as in **a**. **c** Quantification of the duration of mitosis of wt and *BRCA2*^*–/–*^ DLD-1 cells treated with 200 nM alisertib or untreated. The median with interquartile range is depicted. Significance was tested with a Kruskal–Wallis test with Dunn’s multiple comparisons, *p ≤ 0.05, **p ≤ 0.01, ***p ≤ 0.001, ns not significant. **d** Percentages of wt and *BRCA2*^*–/–*^ DLD-1 cells treated with 200 nM alisertib or not, with mitotic aberrations. **e** Percentages of wt and *BRCA2*^*–/–*^ DLD-1 cells treated with 200 nM alisertib or left untreated, that undergo cell death (Kruskal–Wallis test with Dunn’s multiple comparisons, *p ≤ 0.05, **p ≤ 0.01, ***p ≤ 0.001, ns not significant). **f** Pie charts with the different types of mitotic aberrations in wt and *BRCA2*^*–/–*^ DLD-1 cells treated with 200 nM alisertib. **g** BT-549-shBRCA2^dox^ cells were pretreated for 3 days with doxycycline and treated with alisertib for an additional 2 days. Cells were single-cell sorted and whole-genome sequenced. The number of focal aberrations and the number of whole-chromosome aberrations per cell were counted. Medians with interquartile range are depicted and statistical analyses were performed using a Kruskal–Wallis test with Dunn’s multiple comparisons, *p ≤ 0.05, **p ≤ 0.01, ***p ≤ 0.001, ns not significant

In order to investigate the cell fate of BRCA2-inactivated cells upon Aurora-A inhibition, we analyzed *BRCA2*^–/–^ and *BRCA2* wt DLD-1 cells using live-cell microscopy (Fig. 6a). As expected, alisertib treatment resulted in reduced phosphorylation of HH3, and an increased number of mitotic *BRCA2* wt DLD-1 cells (Suppl. Figure 6a). Treatment with alisertib increased mitotic duration in *BRCA2* wt cells (*p* < 0.01) and to a greater extent in *BRCA2*^–/–^ (*p* < 0.001) DLD-1 cells compared to untreated *BRCA2* wt and *BRCA2*^–/–^ DLD-1 cells (Fig. 6a–c). Also, the amount of mitotic aberrations increased upon alisertib treatment. While in alisertib-treated *BRCA2* wt DLD-1 cells, 19% of the mitoses were aberrant, alisertib-treated *BRCA2*^–/–^ DLD-1 cells displayed aberrations in 85% of the mitoses (*p* < 0.001) (Fig. 6d). Aberrant mitoses led to cell death in 48.0% of *BRCA2*^–/–^ cells compared to 19.4% in *BRCA2* wt DLD-1 cells treated with alisertib (*p* < 0.001) (Fig. 6e). Of note, the majority of aberrant mitoses in *BRCA2*^–/–^ cells treated with alisertib involved cytokinesis failure (54.5% in *BRCA2*^–/–^ compared to 6.4% in wt DLD-1 cells) (Fig. 6f). Notably, when alisertib was tested on non-transformed MCF10A cells, we did not observe pronounced cell death, but did detect nuclear abnormalities (Suppl. Figure 6d, left panels), and arrested proliferation (Suppl. Figure 6d, right panel).

Collectively, our results suggest that BRCA2-inactivated cells are more sensitive to inactivation of the TPX2/Aurora-A kinase signaling axis compared to BRCA2-proficient cells, which is likely attributed to an increase in mitotic aberrations.

To further substantiate our findings, we analyzed the effects of BRCA2 inactivation and alisertib treatment at the genomic level. To this end, we used single-cell whole-genome sequencing (Fig. 6g, Suppl. Fig. 7). As expected, BRCA2 inactivation leads to increased numbers of focal copy-number alterations (Fig. 6g, left panel), whereas alisertib treatment resulted in whole-chromosome aneuploidies (Fig. 6g, right panel). Notably, the increased degree of whole-chromosome aneuploidies was not observed in cells with combined alisertib treatment and BRCA2 depletion, which was very likely a consequence of these cells dying. Remarkably, alisertib treatment also resulted in increased numbers of focal copy-number alterations (Fig. 6g, left panel). These aberrancies may reflect DNA damage that arises as a consequence of aberrant mitoses [19, 20], which may be specifically toxic in HR-deficient cancer cells.

## Discussion

Out of the 11 genes that were identified as part of a co-functionality cluster, we focused on TPX2. Previously, TPX2 was shown to be amplified in a number of genomically unstable cancers, including gastric, colon, oral squamous cell carcinoma, and ovarian cancer [21]. Beyond *TPX2* amplification, elevated protein levels of TPX2 are frequently reported in cancer [22–24], although these effects may be indirectly caused by high levels of proliferation, since TPX2 is post-translationally regulated during the cell cycle [25]. Regardless of whether TPX2 overexpression is caused by the underlying amplification, overexpression of TPX2 positively correlates with tumor grade, stage, lymph node metastasis, remote metastasis, recurrence, and a poor prognosis and poor patient survival [22, 23, 26]. These observations are in line with genomically unstable tumors that are more often high-grade tumors with a poor prognosis [2, 27].

Our results indicate that cancer cells, especially those with defective HR, increasingly depend on the presence of TPX2, or its associated kinase Aurora-A, for their survival. *AURKA* still ranked within the top 15% of genes positively associated with genomic instability at place 2012. *TPX2* ranked clearly higher, which is in line with a previous study, in which *TPX2* but not *AURKA* was part of the CIN70 set of genes which is positively associated with chromosomal instability (CIN) [28]. These differences likely represent alternative means by which gene activation is achieved in cancer cells, or differential requirements for TPX2 and Aurora-A in achieving elevated Aurora-A kinase activity.

In this study, HR inactivation was modeled through BRCA2 inactivation. In line with this, a single-nucleotide polymorphism (SNP) near the *AURKA* gene was previously associated with cancer risk in BRCA2 mutation carriers [29]. Yet, in our FGmRNA analysis, genomically unstable samples were included regardless of the underlying gene mutations. It is therefore unlikely that sensitivity toward TPX2 or Aurora-A inactivation is restricted to BRCA2-defective cells. Very likely, *TPX2/AURKA* overexpression or gene amplifications are only allowed in specific genetic contexts. It would be interesting to further investigate which genetic aberrations co-occur with *TPX2/AURKA*, to further guide the clinical implementation of Aurora kinase inhibitors.

One possible explanation for the observed dependence of BRCA2-inactivated cells on TPX2 could be that TPX2 is required for residual DNA repair in HR-deficient cells, for instance, through non-homologous end joining (NHEJ). However, we did not observe altered kinetics of DNA damage clearance of IR-induced DNA damage. These observations are in good agreement with previously published genome-wide shRNA screens for DNA repair regulators [30, 31]. In these studies, loss of canonical NHEJ regulators (including *PRKDC* and *XRCC4* and *XRCC5*) resulted in compensation through elevated HR, which was not observed upon TPX2 or Aurora-A depletion (Suppl. Figure 3g). These data suggest that increased dependence on TPX2 or Aurora-A is not due to a role for TPX2 or Aurora-A in DNA repair.

We did observe a small but statistically significant increase in the amount of DNA breaks in TPX2-depleted cells (Fig. 3d), and increased numbers of structural genomic aberrations upon alisertib treatment (Fig. 6g). Inhibition of Aurora-A was previously shown to cause mitotic aberrations, including delayed mitotic entry, multipolar spindles, and defective cytokinesis, which leads to ensuing aneuploidy [32, 33]. Importantly, mitotic failure was shown to cause DNA damage [19, 20]. Such ensuing DNA lesions after mitotic defects may cause DNA replication defects in the subsequent round of cell division, and lead to a mitotic catastrophe in HR-deficient cells [34–36].

Alternatively, the high proliferation rates and frequently compromised DNA repair in genomically unstable cancer cells, may make these cells increasingly dependent on mitotic processes in general for faithful cell division. Notably, also BRCA2 has been implicated in regulating mitotic progression, specifically in cytokinesis [37], and BRCA1 has been demonstrated to bind and control Aurora-A [38]. This may lead to a general dependence on mitotic regulators. Yet, our original 11-gene cluster contained multiple mitotic regulators, of which only two genes (*TPX2* and *KIF2C*) showed differential effects in BRCA2-proficient vs BRCA2-deficient cells. Also, while Aurora-A inhibition preferentially affected BRCA2-depleted cells, Aurora-B inhibition did not (Suppl. Figure 6c). Future research is warranted to test whether other components of the mitotic spindle or spindle assembly checkpoint are conditionally required in BRCA2-defective cells. In this context, especially MAD2 and CDC20 are interesting, as their expression is highly correlated to TPX2 levels [39].

Although no chemical TPX2 inhibitors are currently available, Aurora-A inhibitors have been extensively studied. Notably, cancers driven by *MYC* amplification were shown to be selectively sensitive to Aurora-A inhibition [32, 40, 41]. Specifically, *AURKA* and *TPX2* together with *MYC* appear to act as driver genes in *MYC*-driven cancers [40]. Because *TP53* loss and *MYC* amplification is often observed in genomically unstable cancers [4, 21], Aurora-A inhibitors may offer therapeutic benefit to patients with genomically unstable cancers.

The Aurora-A inhibitor alisertib (also known as MLN8237) has been tested preclinically and clinically, either alone or in combination therapy [32, 41]. Especially in combination therapy with spindle poisons such as taxanes, alisertib induces antitumor effects, for instance, in in vivo TNBC models [42]. Our analysis of MCF10A cells showed that non-transformed cells do display nuclear abnormalities and arrested proliferation in response to alisertib treatment, which warrants an assessment of long-term effects on normal tissues. Clinically, antitumor effects of alisertib were reported in solid and hematological cancers [43–45], and long-term progression-free survival or complete response was observed in some cases in patients with solid cancers or in patients with recurrent ovarian cancer [46, 47].

Notably, inhibition of Aurora-A and CHEK1, a DNA damage checkpoint kinase, showed synergistic effects in vitro, which was attributed to a G_2_/M-phase cell cycle arrest and a consequent increase in apoptosis [48]. Similarly, combined treatment of alisertib with platinum-based drugs appeared particularly beneficial in platinum-resistant recurrent ovarian cancers and small-cell lung cancers [45, 49]. Interestingly, expression of Aurora-A is positively associated with resistance to cisplatin-based chemotherapeutic agents [50]. Possibly, Aurora-A is required to allow cancer cells to proliferate, despite the presence of DNA lesions. Such DNA lesions may be introduced through genotoxic agents and result from DNA repair defects, for instance caused by BRCA2 inactivation. Of note, Aurora-A in conjunction with Polo-like kinase-1 was previously shown to be required to allow cells to restart cell division in situations of DNA damage [51, 52]. In this context, it is of interest to determine whether Aurora-A inhibition may potentiate PARP inhibitor treatment in HR-defective tumors. PARP inhibition in HR-defective cancer cells increases the load of DNA lesions [53, 54], which are to a significant degree transmitted into mitosis [34]. In this context, Aurora-A inhibition may affect such tumor cells both at the G_2_/M-phase transition as well as during mitotic progression.

## Materials and methods

### Co-functionality analysis

Using a previously published gene co-regulation network (available at http://genenetwork.nl), networks of genes that show a strong predicted co-functionality are constructed. The likelihood for an individual gene to be part of a biological pathway (i.e., gene set) is described by a Spearman correlation coefficient. Based on the co-regulated gene network, we calculated the correlation coefficients per individual gene with every gene set as defined in a selected database (Gene Ontology, KEGG, Reactome, or Biocarta). This resulted in a vector of *n* correlation coefficients (i.e., functional likelihood vector) for each individual gene. The number of gene sets in the selected database determines the number *n*. Subsequently, the correlation between functional likelihood vectors of individual genes was calculated (i.e., co-functionality correlation). A high co-functionality correlation indicates that two individual genes have similar predicted biological functions. Genes are plotted in the co-functionality network when the co-functionality correlation is above a predefined threshold (correlation coefficient > 0.5).

### Cell lines

Human breast cancer cell lines BT-549, MDA-MB-231, HCC38, and HCC1806 were obtained from ATCC (#HTB122, #HTB26, #CRL2314, and #CRL2335) and SUM149 was obtained from Asterand Bioscience. BT-549, HCC38, and HCC1806 were maintained in RPMI medium (Invitrogen), supplemented with 10% fetal calf serum (FCS). Non-transformed human MCF10A breast epithelial cells (Crl-10317), HeLa human cervical cancer cells (#CCL2), and HEK293T (#CRL3216) human embryonic kidney were obtained from ATCC. MDA-MB-231 cells were cultured in DMEM medium (Invitrogen), supplemented with 10% FCS. SUM149 was cultured in Ham/F-12 (1:1) medium (Invitrogen), supplemented with 10% FCS, 1 μg/mL hydrocortisone, and 5 μg/mL insulin (Sigma). DLD-1 human colorectal adenocarcinoma cells were described previously [34], and cultured in RPMI with 10% FCS. All cell lines were cultured in a humidified incubator at 37 °C and 5% CO_2_. Mouse mammary tumor cell lines K14-Cre;*Brca2*^wt/wt^;*p53*^F2-10/F2-10^, K14-Cre;*Brca2*^F11/F11^;*p53*^F2-10/F2-10^, and K14-Cre;*Brca2*^F11/F11^;*p53*^F2-10/F2-10^ + iBac-*Brca*2 were described previously [55–57] and were maintained in DMEM/F-12 medium (1:1 ratio, Invitrogen), supplemented with 10% FCS, penicillin (50 U/mL, Invitrogen), streptomycin (50 μg/mL, Invitrogen), insulin (5 μg/mL, Sigma), epidermal growth factor (5 ng/mL, Preprotech), and cholera toxin (5 ng/mL, Sigma) at 37 °C under hypoxic conditions (1% O_2_ and 5% CO_2_). Cells were plated 24 h prior to any treatment or transfection. All cell lines were routinely tested for mycoplasma infection and authenticated by STR profiling.

### Virus infections

To establish BT-549, MDA-MB-231, SUM149, HCC38, and HCC1806 cell lines stably expressing doxycycline-inducible shRNAs, cells were infected with lentiviral particles as described previously [58]. In short, HEK293T cells were transfected with 10 μg of pLKO-Tet-Puro-BRCA2 (5′-AACAACAATTACGAACCAAACTT-3′), in combination with 4 μg of delta YPR, 2.6 μg of VSV-G, and 1.6 μg of pAdvantage (Promega). After transfection, supernatant medium containing virus particles was filtered and transferred to recipient cells in three subsequent 12-h periods. Infected cells were selected using puromycin (1 µg/mL, Sigma). To induce shRNA expression, shBRCA2-recipient cells were treated with doxycycline (1 µg/mL) for 48 or 72 h.

To generate cell lines overexpressing TPX2, human TPX2 was amplified using pmCherry-TPX2 as a template, which was a kind gift from Patricia Wadsworth (Addgene plasmid #31227) [59]. The TPX2 transcript was PCR amplified using the following primers: forward: (5′-GATCCATGAAAGTTTCTAACAACAAA-3′) and reverse (5′-AATTCAAAAAATGAAAGTTTCTAACAACAAA-3′). The resulting PCR product was cloned into pBabe-hygro using the *Bam*HI and *Eco*RI restriction sites. The pBabe-hygro plasmid was a kind gift from Hartmut Land and Jay Morgenstern and Bob Weinberg (Addgene plasmid #1765). The resulting plasmid was verified using Sanger sequencing using the following primer: (5′-GAAATTTGTGATGCTATTGC-3′). Subsequently, BT-549-shBRCA2^dox^ [60] cells were retrovirally infected with pBabe-TPX2 or pBabe-EV (empty vector). To this end, HEK293T cells were transfected with 10 μg of pBabe-EV or with pBabe-TPX2 combined with 2.5 μg of pMD/p and 7.5 μg of pMDg packaging plasmids as described previously [61]. The supernatant was collected, filtered, and transferred to BT-549-shBRCA2^dox^ cells in three subsequent 12-h periods. Recipient cells were selected with hygromycin (200 μg/mL, Sigma).

### RNA interference

Two independent Stealth siRNA targeting sequences (ThermoFisher Scientific) were used for the following genes: *BIRC5, UBE2C, CENPA, TPX2, KIF2C, DEK, CDCA3, SKP2, RAD21, MYBL2*, and *WDR67* (Table 1). For each transfection, Stealth siRNA negative control (ctrl) scrambled sequences were taken along. Cells were transfected with siRNAs (final concentration of 133 nM) in Opti-MEM (Life Technologies) at 80–90% confluency using Oligofectamine (Invitrogen). Forty-eight hours after transfection, cells were trypsinized and counted using a counting chamber and were replated at 30,000 cells per well in a six-well plate in the presence or absence of doxycycline (1 µg/mL). After 5 days, cells were trypsinized and the amounts of living cells per well were counted.

**Table 1:** siRNA sequences

Genes	siRNA sequence 1: 5′–3′	siRNA sequence 2: 5′–3′
*BIRC5*	GAGGCTGGCTTCATCCACT	GGACCACCGCATCTCTACA
*UBE2C*	CAGCAGGAGCTGATGACCCTCATG	GAAGTACCTGCAAGAAACCTACTCA
*CENPA*	ACAGTCGGCGGAGACAAGG	TCATAGAAGATGTATCATA
*TPX2*	ATGAAAGTTTCTAACAACAAA	AAGAATGGAACTGGAGGGCTT
*KIF2C*	GAGAAGAAGGCCCAGAACT	CCAACGCAGTAATGGTTTA
*DEK*	CAAAGTATCTGGAGAACCA	CCATTGCCGAAATCTAAAA
*CDCA3*	CGCAATAGATGGAAACCAA	GTATTGCACGGACACCTAT
*SKP2*	AGGTCTCTGGTGTTTGTAA	GACCTATCGAACTCAGTTA
*RAD21*	CTCCAAATATCTGTCAGCTAA	GAACCGTACAGTGACATCA
*MYBL2*	CCCTGTCAGGTATCAAAGA	CCACATCGAAGGAACAGGA
*WDR67(1)*	GCGTTTAGTACCCTCATAGAT	GGGACGGAATTATAGTGAACA
*WDR67(2)*	GCCTCTGCTCAGCTGTAATCT	AATTATAGTGAACATTATTC
*WDR67(3)*	GTTTTCTTTCTACCATTAAG	
*AURKA*	AUUCUUCCCAGCGCGUUCC	AUGCCCUGUCUUACUGUCA

### Immunofluorescence microscopy

Doxycycline-inducible cell lines were grown on coverslips and treated with doxycycline (1 µg/mL) for 3 days or cells were left untreated. If indicated, cells were irradiated (5 Gy) using a CIS international/IBL 637 cesium^137^ source (dose rate: 0.010124 Gy/s). After 3 h of irradiation, cells were fixed with 2% paraformaldehyde, permeabilized in 0.1% Triton X-100 in PBS, blocked in 4% bovine serum albumin (BSA) in PBS, and incubated with primary antibodies against RAD51 (GeneTex, #gtx70230, 1:400) and γH2AX (Cell Signaling, #9718, 1:200). Secondary antibodies Alexa-488 or Alexa-647 (1:500) were used and slides were stained with DAPI. Images were made using a Leica DM6000B microscope with a ×63 immersion objective.

### Western blotting

Cells were lysed in ice-cold M-Per lysis buffer (Pierce), complemented with 1% protease and 1% phosphatase inhibitor cocktail (Roche). Proteins were then separated on SDS-polyacrylamide gels (SDS-PAGE) and electrophoretically transferred onto a PVDF membrane (Millipore). Membranes were blocked with 5% skimmed milk (Sigma) in Tris-buffered saline (TBS) with 0.05% Tween-20 (Sigma) and probed with specific antibodies recognizing BRCA2 (Millipore, OP95, 1:1000), TPX2 (Novus Biologicals, NB500-179, 1:1000), Aurora-A (Abcam, ab13824, 1:1000), Aurora-A (phospho-Thr288)/Aurora-B (phospho-Thr232)/Aurora-C (phospho-Thr198) (Cell Signaling#2914), or β-actin (MP Biomedicals, #69100, 1:2000). Horseradish peroxidase-conjugated secondary antibodies (DAKO) were diluted 1:2000 and were visualized using chemiluminescence (Lumi-Light, Roche Diagnostics) on a Bio-Rad bioluminescence device. Images were made using Quantity One/ChemiDoc XRS software (Bio-Rad).

### Flow cytometry

Cells were fixed in ice-cold 70% ethanol, and incubated overnight at 4 °C. Subsequently, cells were stained for rabbit anti-phospho-histone H3 antibody (1:100, Cell Signalling, #9701) and mouse MPM2 (1:100, Millipore, 05-368). After washing with PBS–0.05% Tween-20, cells were incubated with Alexa 488-conjugated and Alexa 647-conjugated secondary antibodies (1:100, Molecular Probes) and counterstained with propidium iodide/RNase (Sigma). FACS analyses were performed on a FACS-Calibur (Becton Dickinson) and samples were analyzed using Cell Quest software. Data were analyzed using FlowJo software. At least 10,000 events were analyzed per sample.

### cDNA synthesis and qRT-PCR

RNA was isolated from frozen cell pellets using the RNeasy kit (Qiagen). Between 100 ng and 1 μg of total RNA was reverse transcribed into cDNA using Superscript III (Bio-Rad). The resulting first-strand cDNA was used as a template in the qRT-PCR. Samples were amplified using the indicated primers (Table 2), cDNA, and a SYBR Green master mix (Bio-Rad).

**Table 2:** qRT-PCR primers

Genes	Forward primer 5′–3′	Reverse primer 5′–3′
*BIRC5*	TCAAGGACCACCGCATCTCTA	TGAAGCAGAAGAAACACTGGGC
*UBE2C*	TGATGTCTGGCGATAAAGGGATT	GTGATAGCAGGGCGTGAGGAA
*CENPA*	CTTCCTCCCATCAACACAGTC	TGCTTCTGCTGCCTCTTGTAG G
*TPX2*	CGAAAGCATCCTTCATCTCC	TCCTTGGGACAGGTTGAAAG
*KIF2C*	ACTCTAGGACTTGCATGATTGCC	TGGGTGT CAAACCAAACAGA
*DEK*	TGTTAAGAAAGCAGATAGCAGCACC	ATTAAAGGTTCATCATCTGAACTATCCTC
*CDCA3*	TGGTATTGCAC GGACACCTA	TGTTTCACCAGTG GGCTTG
*MYBL2*	GATTCCTGTAACAGCCTCAC	CCAGAAGTTCAGAAACTGGG
*SKP2*	GGTGTTTGTAAGAGGTGGTATCGC	CACGAAAAGGGCTGAAATGTTC
*RAD21*	CCTCAGCAGGTAGAGCAAATGG	GCATCTGCTGAGTGCGTTTGTT
*WDR67*	GCGGGACGGAATTATAGTG	GCTGAACAAGATTGAACCTG
*GAPDH*	CACCACCATGGAGAAGGCTGG	CCAAAGTTGTCATGGATGACC
*BRCA2*	TTTTTAGATCCAGACTTTCAGC	TGGATCTGAGCTTGTTTCTT

### Clonogenic and short-term survival assays

For clonogenic survival assays, cells were cultured in six-well plates. When colonies reached a size of approximately 50 cells, typically after 10–14 days, colonies were washed with PBS, fixed with methanol, and subsequently stained with Coomassie Brilliant Blue (CBB). Amounts of surviving colonies in drug-treated samples were normalized to DMSO-treated samples. For short-term survival assays, cells were treated with alisertib with indicated concentrations for 4 days after which methyl-thiazol-tetrazolium (MTT) (5 mg/mL) was added. After 4 h, the medium was removed and DMSO was added to dissolve formazan crystals. Absorbance values were measured on a Bio-Rad benchmark III Biorad spectrophotometer and they were normalized to DMSO absorbance values.

### Live-cell microscopy

BT-549 cells were transduced with H2B-EGFP as previously described [34], and plated in eight-chambered cover glass plates (Lab-Tek-II, Nunc). Doxycycline-inducible cells were treated with doxycycline for 24 h to induce BRCA2 depletion prior to live-cell microscopy. Cells were followed using a DeltaVision Elite microscope with a ×20 objective. Cells were tracked for 60–65 h or until they migrated out of frame. Every 6 min, six images were taken in the *z*-axis with an interval of 0.5 μm. Time-lapse images were made and analyzed using SoftWorX software (Applied Precision/GE Healthcare). Duration of mitosis was quantified from prometaphase to anaphase. Aberrant mitoses included unequal chromosome segregation, absence of a bipolar spindle, or cytokinesis failure.

### Single-cell whole-genome analysis

BT-549-shBRCA2#2^dox^ cells were treated with doxycycline (1 μg/mL) or alisertib (200 nM) as indicated, and after 72 h, cells were single-cell sorted into 96-well plates (48 cells per condition), using a Hoechst/propidium iodide double staining. Only G1 cells were included. Cells were then lysed, and DNA was sheared. DNA was barcode-labeled, followed by library preparation as described previously [62], in an automated fashion using an Agilent Bravo robot. Single-cell libraries were pooled and analyzed on an Illumina Hiseq2500 sequencer. Sequencing data was analyzed using AneuFinder software as described previously [63]. Focal deviations and whole-chromosome deviations from the modal state in control-treated BT-549 samples were analyzed.

## Electronic supplementary material

Supplemental Figure Legends

Supplemental Figure 1

Supplemental Figure 2

Supplemental Figure 3

Supplemental Figure 4

Supplemental Figure 5

Supplemental Figure 6

Supplemental Figure 7

## References

[1] Buccitelli C, Salgueiro L, Rowald K, Sotillo R, Mardin BR, Korbel JO (2017). Pan-cancer analysis distinguishes transcriptional changes of aneuploidy from proliferation. Genome Res.

[2] Fehrmann RSN, Karjalainen JM, Krajewska M, Westra HJ, Maloney D, Simeonov A (2015). Gene expression analysis identifies global gene dosage sensitivity in cancer. Nat Genet.

[3] Shah SP, Roth A, Goya R, Oloumi A, Ha G, Zhao Y (2012). The clonal and mutational evolution spectrum of primary triple-negative breast cancers. Nature.

[4] Cancer Genome Atlas Network. (2012). Comprehensive molecular portraits of human breast tumours. Nature.

[5] Ashworth A, Lord CJ, Reis-Filho JS (2011). Genetic interactions in cancer progression and treatment. Cell.

[6] Engebraaten O, Vollan HKM, Børresen-Dale AL (2013). Triple-negative breast cancer and the need for new therapeutic targets. Am J Pathol.

[7] Aguilera A, Gómez-González B (2008). Genome instability: a mechanistic view of its causes and consequences. Nat Rev Genet.

[8] Davies H, Glodzik D, Morganella S, Yates LR, Staaf J, Zou X (2017). HRDetect is a predictor of BRCA1 and BRCA2 deficiency based on mutational signatures. Nat Med.

[9] Roy R, Chun J, Powell SN (2012). BRCA1 and BRCA2: important differences with common interests. Nat Rev Cancer.

[10] Jasin M, Rothstein R (2013). Repair of strand breaks by homologous recombination. Cold Spring Harb Perspect Biol.

[11] Moynahan ME, Jasin M (2010). Mitotic homologous recombination maintains genomic stability and suppresses tumorigenesis. Nat Rev Mol Cell Biol.

[12] Ludwig T, Chapman DL, Papaioannou VE, Efstratiadis A (1997). Targeted mutations of breast cancer susceptibility gene homologs in mice: lethal phenotypes of Brca1, Brca2, Brca1/Brca2, Brca1/p53, and Brca2/p53 nullizygous embryos. Genes Dev.

[13] Suzuki A, la Pompa de JL, Hakem R, Elia A, Yoshida R, Mo R (1997). Brca2 is required for embryonic cellular proliferation in the mouse. Genes Dev.

[14] Hakem R, la Pompa de JL, Sirard C, Mo R, Woo M, Hakem A (1996). The tumor suppressor gene Brca1 is required for embryonic cellular proliferation in the mouse. Cell.

[15] Luo J, Solimini NL, Elledge SJ (2009). Principles of cancer therapy: oncogene and non-oncogene addiction. Cell.

[16] Weinstein IB (2002). CANCER: enhanced: addiction to oncogenes--the Achilles heal of cancer. Science.

[17] Kufer TA, Silljé HHW, Körner R, Gruss OJ, Meraldi P, Nigg EA (2002). Human TPX2 is required for targeting Aurora-A kinase to the spindle. J Cell Biol.

[18] Tayyar Y, Jubair L, Fallaha S, McMillan NAJ (2017). Critical risk-benefit assessment of the novel anti-cancer Aurora A kinase inhibitor alisertib (MLN8237): a comprehensive review of the clinical data. Crit Rev Oncol Hematol.

[19] Janssen A, van der Burg M, Szuhai K, Kops GJ, Medema RH (2011). Chromosome segregation errors as a cause of DNA damage and structural chromosome aberrations. Science.

[20] Crasta K, Ganem NJ, Dagher R, Lantermann AB, Ivanova EV, Pan Y (2012). DNA breaks and chromosome pulverization from errors in mitosis. Nature.

[21] Ciriello G, Miller ML, Aksoy BA, Senbabaoglu Y, Schultz N, Sander C (2013). Emerging landscape of oncogenic signatures across human cancers. Nat Genet.

[22] Tomii C, Inokuchi M, Takagi Y, Ishikawa T, Otsuki S, Uetake H (2017). TPX2 expression is associated with poor survival in gastric cancer. World J Surg Oncol.

[23] Wei P, Li D, Xu Y, Cai S (2013). Validation of TPX2 as a novel prognostic marker for malignant progression and metastasis of colon cancer. J Clin Oncol.

[24] Shigeishi H, Fujimoto S, Hiraoka M, Ono S, Taki M, Ohta K (2009). Overexpression of the receptor for hyaluronan-mediated motility, correlates with expression of microtubule-associated protein in human oral squamous cell carcinomas. Int J Oncol.

[25] Perez de Castro I, Malumbres M (2013). Mitotic stress and chromosomal instability in cancer: the case for TPX2. Genes Cancer.

[26] Cáceres-Gorriti KY, Carmona E, Barrès V, Rahimi K, Létourneau IJ, Tonin PN (2014). RAN nucleo-cytoplasmic transport and mitotic spindle assembly partners XPO7 and TPX2 are new prognostic biomarkers in serous epithelial ovarian cancer. PLoS ONE.

[27] Habermann JK, Doering J, Hautaniemi S, Roblick UJ, Bündgen NK, Nicorici D (2009). The gene expression signature of genomic instability in breast cancer is an independent predictor of clinical outcome. Int J Cancer.

[28] Carter SL, Eklund AC, Kohane IS, Harris LN, Szallasi Z (2006). A signature of chromosomal instability inferred from gene expression profiles predicts clinical outcome in multiple human cancers. Nat Genet.

[29] Blanco I, Kuchenbaecker K, Cuadras D, Wang X, Barrowdale D, de Garibay GR (2015). Assessing associations between the AURKA-HMMR-TPX2-TUBG1 functional module and breast cancer risk in BRCA1/2 mutation carriers. PLoS ONE.

[30] Słabicki M, Theis M, Krastev DB, Samsonov S, Mundwiller E, Junqueira M (2010). A genome-scale DNA repair RNAi screen identifies SPG48 as a novel gene associated with hereditary spastic paraplegia. PLoS Biol.

[31] Adamson B, Smogorzewska A, Sigoillot FD, King RW, Elledge SJ (2012). A genome-wide homologous recombination screen identifies the RNA-binding protein RBMX as a component of the DNA-damage response. Nat Cell Biol.

[32] Niu H, Manfredi M, Ecsedy JA (2015). Scientific rationale supporting the clinical development strategy for the investigational aurora A kinase inhibitor alisertib in cancer. Front Oncol.

[33] Sloane DA, Trikic MZ, Chu MLH, Lamers MB, Mason CS, Mueller I (2010). Drug-resistant Aurora A mutants for cellular target validation of the small molecule kinase inhibitors MLN8054 and MLN8237. ACS Chem Biol.

[34] Schoonen PM, Talens F, Stok C, Gogola E, Heijink AM, Bouwman P (2017). Progression through mitosis promotes PARP inhibitor-induced cytotoxicity in homologous recombination-deficient cancer cells. Nat Commun.

[35] Mankouri HW, Huttner D, Hickson ID (2013). How unfinished business from S-phase affects mitosis and beyond. EMBO J.

[36] Castedo M, Perfettini JL, Roumier T, Andreau K, Medema R, Kroemer G (2004). Cell death by mitotic catastrophe: a molecular definition. Oncogene.

[37] Daniels MJ (2004). Abnormal cytokinesis in cells deficient in the breast cancer susceptibility protein BRCA2. Science.

[38] Ertych N, Stolz A, Valerius O, Braus GH, Bastians H (2016). CHK2– BRCA1 tumor-suppressor axis restrains oncogenic Aurora-A kinase to ensure proper mitotic microtubule assembly. Proc Natl Acad Sci USA.

[39] Borges D, de P, Santos dos AWA, Paier CRK, Júnior RibeiroHL, Costa MB, Farias IR (2018). Prognostic importance of Aurora Kinases and mitotic spindle genes transcript levels in Myelodysplastic syndrome. Leuk Res.

[40] Takahashi Y, Sheridan P, Niida A, Sawada G, Uchi R, Mizuno H (2015). The AURKA/TPX2 axis drives colon tumorigenesis cooperatively with MYC. Ann Oncol.

[41] Dauch D, Rudalska R, Cossa G, Nault JC, Kang TW, Wuestefeld T (2016). A MYC–Aurora kinase A protein complex represents an actionable drug target in p53-altered liver cancer. Nat Med.

[42] Huck JJ, Zhang M, Mettetal J, Chakravarty A, Venkatakrishnan K, Zhou X (2014). Translational exposure-efficacy modeling to optimize the dose and schedule of taxanes combined with the investigational Aurora A kinase inhibitor MLN8237 (alisertib). Mol Cancer Ther.

[43] Barr PM, Li H, Spier C, Mahadevan D, LeBlanc M, Ul Haq M (2015). Phase II intergroup trial of alisertib in relapsed and refractory peripheral T-cell lymphoma and transformed mycosis fungoides: SWOG 1108. J Clin Oncol.

[44] Dickson MA, Mahoney MR, Tap WD, D’Angelo SP, Keohan ML, Van Tine BA (2016). Phase II study of MLN8237 (Alisertib) in advanced/metastatic sarcoma. Ann Oncol.

[45] Melichar B, Adenis A, Lockhart AC, Bennouna J, Dees EC, Kayaleh O (2015). Safety and activity of alisertib, an investigational Aurora kinase A inhibitor, in patients with breast cancer, small-cell lung cancer, non-small-cell lung cancer, head and neck squamous-cell carcinoma, and gastro-oesophageal adenocarcinoma: a five-arm phase 2 study. Lancet Oncol.

[46] Coleman R, Roszak A, Behbakht K, Ray-Coquard IL, Matulonis U, Liu H (2014). 876orandomized phase 2 study of investigational, selective aurora a kinase inhibitor alisertib (mln8237) with weekly paclitaxel vs paclitaxel alone in patients (pts) with recurrent ovarian cancer (oc). Ann Oncol.

[47] Graff JN, Higano CS, Hahn NM, Taylor MH, Zhang B, Zhou X (2016). Open-label, multicenter, phase 1 study of alisertib (MLN8237), an Aurora A kinase inhibitor, with docetaxel in patients with solid tumors. Cancer.

[48] Alcaraz-Sanabria A, Nieto-Jiménez C, Corrales-Sánchez V, Serrano-Oviedo L, Andrés-Pretel F, Montero JC (2017). Synthetic lethality interaction between aurora kinases and CHEK1 inhibitors in ovarian cancer. Mol Cancer Ther.

[49] Matulonis UA, Sharma S, Ghamande S, Gordon MS, Del Prete SA, Ray-Coquard I (2012). Phase II study of MLN8237 (alisertib), an investigational Aurora A kinase inhibitor, in patients with platinum-resistant or -refractory epithelial ovarian, fallopian tube, or primary peritoneal carcinoma. Gynecol Oncol.

[50] Xu J, Yue CF, Zhou WH, Qian YM, Zhang Y, Wang SW (2014). Aurora-A contributes to cisplatin resistance and lymphatic metastasis in non-small cell lung cancer and predicts poor prognosis. J Transl Med.

[51] van Vugt MA, Bràs A, Medema RH (2004). Polo-like kinase-1 controls recovery from a G2 DNA damage-induced arrest in mammalian cells. Mol Cell.

[52] Macurek L, Lindqvist A, Lim D, Lampson MA, Klompmaker R, Freire R (2008). Polo-like kinase-1 is activated by Aurora A to promote checkpoint recovery. Nature.

[53] Farmer H, McCabe N, Lord CJ, Tutt ANJ, Johnson DA, Richardson TB (2005). Targeting the DNA repair defect in BRCA mutant cells as a therapeutic strategy. Nature.

[54] Bryant HE, Schultz N, Thomas HD, Parker KM, Flower D, Lopez E (2005). Specific killing of BRCA2-deficient tumours with inhibitors of poly(ADP-ribose) polymerase. Nature.

[55] Evers B, Drost R, Schut E, de Bruin M, van der Burg E, Derksen PWB (2008). Selective inhibition of BRCA2-deficient mammary tumor cell growth by AZD2281 and cisplatin. Clin Cancer Res.

[56] Rottenberg S, Jaspers JE, Kersbergen A, van der Burg E, Nygren AOH, Zander SAL (2008). High sensitivity of BRCA1-deficient mammary tumors to the PARP inhibitor AZD2281 alone and in combination with platinum drugs. Proc Natl Acad Sci USA.

[57] Liu X, Holstege H, van der Gulden H, Treur-Mulder M, Zevenhoven J, Velds A (2007). Somatic loss of BRCA1 and p53 in mice induces mammary tumors with features of human BRCA1-mutated basal-like breast cancer. Proc Natl Acad Sci USA.

[58] Heijink AM, Blomen VA, Bisteau X, Degener F, Matsushita FY, Kaldis P (2015). A haploid genetic screen identifies the G1/S regulatory machinery as a determinant of Wee1 inhibitor sensitivity. Proc Natl Acad Sci USA.

[59] Ma N, Tulu US, Ferenz NP, Fagerstrom C, Wilde A, Wadsworth P (2010). Poleward transport of TPX2 in the mammalian mitotic spindle requires dynein, Eg5, and microtubule flux. Mol Biol Cell.

[60] Morgenstern JP, Land H (1990). Advanced mammalian gene transfer: high titre retroviral vectors with multiple drug selection markers and a complementary helper-free packaging cell line. Nucleic Acids Res.

[61] van Vugt MA, Gardino AK, Linding R, Ostheimer GJ, Reinhardt HC, Ong SE (2010). A mitotic phosphorylation feedback network connects Cdk1, Plk1, 53BP1, and Chk2 to inactivate the G2/M DNA damage checkpoint. PLoS Biol.

[62] van den Bos H, Spierings DCJ, Taudt A, Bakker B, Porubsky D, Falconer E (2016). Single-cell whole genome sequencing reveals no evidence for common aneuploidy in normal and Alzheimer’s disease neurons. Genome Biol.

[63] Bakker B, Taudt A, Belderbos ME, Porubsky D, Spierings DCJ, de Jong TV (2016). Single-cell sequencing reveals karyotype heterogeneity in murine and human malignancies. Genome Biol.

