# Supplementary material for: TPX2/Aurora kinase A signaling as a potential therapeutic target in genomically unstable cancer cells
Source: Oncogene. 2018 Sep 3;38(6):852–67. doi: 10.1038/s41388-018-0470-2 (PMC6367211; doi:10.1038/s41388-018-0470-2)
Supplement: Supplementary file 1 — Supplemental Figure Legends [file 41388_2018_470_MOESM1_ESM.docx]

**Supplemental Figure Legends**

**Supplemental Figure 1: (A)** BT-549-shBRCA2^dox^ cells were left untreated or were treated with doxycycline (3 days) and were transfected with indicated siRNAs. After 48h siRNA transfection, cells were collected and mRNA expression levels of the respective genes were compared to transcript levels of *GAPDH*. Averages and standard deviations of 2 experiments with 3 replicates each are shown. **(B)** BT-549-shBRCA2^dox^ cells were treated with doxycycline (3 days) or left untreated after which cells were stained with propidium iodide and analyzed by flow cytometry. **(C)** BT-549-shBRCA2^dox^ cells were treated with doxycycline (3 days) and were subsequently transfected with indicated siRNAs. 30,000 cells were plated 48h following transfection. Viable cells were counted 5 days later. Each data point represents a cell count replicate (dark gray= untreated, light gray= doxycycline-treated). Orange squares indicate median cell counts of untreated cells, blue squares indicate median cell counts of doxycycline-treated cells. For each condition, at least 2 experimental replicates were performed, with three technical replicates each.

**Supplemental Figure 2: (A)** MB-231-shBRCA2^dox^, SUM149-shBRCA2^dox^, HCC1806-shBRCA2^dox^ and HCC38-shBRCA2^dox^ were treated with doxycycline (3 days) or left untreated and immunoblotted for BRCA2 and HSP90. **(B)** Amounts of viable cells were counted at 5 days after transfection with control siRNA (ctrl), or siRNAs targeting TPX2 or Aurora-A. Each data point represents a cell count replicate (dark gray= untreated, light gray= doxycycline-treated). Orange squares indicate median cell counts of untreated cells, blue squares indicate median cell counts of doxycycline-treated cells. For each condition, at least 2 experimental replicates were performed, with three technical replicates.

**Supplemental Figure 3: (A)** Two western blot replicates of BT-549-shBRCA2^dox^ cells stably infected with pBabe-EV or pBabe-TPX2 and immunoblotted for TPX2 and Actin. Fold change of protein expression is compared to EV and indicated below the blot. **(B)** Percentages of clonogenic cell survival of BT-549-shBRCA2^dox^ pBabe-EV or pBabe-TPX2 cells treated with doxycycline compared to untreated cells (unpaired two-tailed t-test, ns). **(C)** Fold change of cl-caspase 3 staining of doxycycline treated cells versus untreated BT-549-shBRCA2^dox^ pBabe-EV and pBabe-TPX2 cells (unpaired two-tailed t-test, ns=not significant). **(D)** BT-549-shBRCA2^dox^ stably transfected infected with pBabe-EV or pBabe-TPX2 were grown on coverslips and irradiated (IR 5 Gy) or left untreated and stained for RAD51 and γH2AX. Scale bars represent 5μm. **(E)** Percentages of BT-549-shBRCA2^dox^ cells stably infected with pBabe-EV or pBabe-TPX2 and treated with doxycycline or left untreated with <5 RAD51 or with ≥5 RAD51 foci per nucleus after irradiation (5 Gy). Experiment was performed in duplicate (n≥90). **(F)** BT-549 cells were transfected with siTPX2 or control siRNA (CTR) and grown on coverslips for 3 days. Cells were fixed at 0.5h or 6h after irradiation (5 Gy) and stained for 53BP1, γH2AX and DAPI. Representative microscopy pictures are shown. **(G)** Analysis of genome-wide DR-GFP DNA reporter repair screens using shRNA (Adamson *et al*, 2012) and esiRNA-based (Slabicki *et a*l, 2010) were analyzed for indicated genes.

**Supplemental Figure 4: (A)** BT-549-shBRCA2^dox^ stably infected with pBabe-EV or pBabe-TPX2 and H2B-GFP were treated with doxycycline or left untreated and were followed with live-cell microscopy for 65h. Each bar represents a single cell: green bars indicate normal mitoses, blue bars indicate aberrant mitoses, and cell death is indicated with black dots. **(B)** Duration of mitosis of BT-549-shBRCA2^dox^ pBabe-EV or pBabe-TPX2 treated with doxycycline or left untreated. Median with interquartile range is shown (Kruskall-Wallis test with Dunn’s multiple comparisons, *= p≤0.05, **= p≤0.01, ***=p≤0.001, ns=not significant). **(C)** Percentages of cells that result in cell death after mitosis in BT-549-shBRCA2^dox^ pBabe-EV or pBabe-TPX2 cells treated with doxycycline or left untreated (Kruskall-Wallis test with Dunn’s multiple comparisons, *= p≤0.05, **= p≤0.01, ***=p≤0.001, ns=not significant). **(D)** Percentages of cells with mitotic aberrations in BT-549-shBRCA2^dox^ pBabe-EV or pBabe-TPX2 cells treated with doxycycline or left untreated (Kruskall-Wallis test with Dunn’s multiple comparisons, *= p≤0.05, **= p≤0.01, ***=p≤0.001, ns=not significant).

**Supplemental Figure 5: (A)** Mouse mammary tumor cells were grown on coverslips and irradiated (IR, 5 Gy) and stained for RAD51 and γH2AX. Scale bars represent 10μm. **(B)** Percentages of cells with ≥10 RAD51 foci per nucleus (n≥81). **(C)** wt and *BRCA2^-/-^* DLD-1 cells were treated as in panel A. Scale bars represent 5μm. **(D)** Percentages of cells with ≥5 RAD51 foci per nucleus (n≥115). **(E)** Wt and *BRCA2^-/-^* DLD-1 cells were transfected with indicated siRNAs and numbers of viable cells were counted after 5 days. Each data point represents a cell count replicate (dark gray= untreated, light gray = doxycycline-treated). Orange squares depict the median cell counts of wt DLD-1 cells and, blue squares depict the median cell count of *BRCA2^-/-^* DLD-1 cells. For each condition, at least 2 experimental replicates were performed, with three technical replicates. **(F)** Wt DLD-1 cells transfected with siRNA sequences targeting Aurora-A or TPX2 were immunoblotted for TPX2, Aurora-A and actin.

**Supplemental Figure 6:**

**(A)** wt DLD-1 cells were treated with 200nM alisertib or left untreated for 24h after which cells were stained for pHH3 and MPM2 and analyzed by flow cytometry. Percentages of cells stained positive for pHH3 and MPM2 are indicated in the profiles. **(B)** HeLa cells were treated with 100 ng/mL nocodazole ain combination with indicated concentrations of alisertib for 16 hours . Cells were harvested and immunoblotted for pAurora-A, pAurora-B and pAurora-C. **(C)** Left panel: BT-549-shBRCA2^dox^ cells were left treated or were pretreated with doxycycline for 48 hours, and subsequently treated with indicated concentrations of alisertib with or without doxycycline for an additional 3 days. Right panel: BT-549-shBRCA2^dox^ cells were treated similar as in for panel C with indicated concentrations of ZM447439. Dose-response curves depict survival of treated compared to control-treated cells. Means and standard errors of 4 replicates are depicted. **(D)** Left panel: representative microscopy images of BT-549-shBRCA2^dox^ cells pretreated with doxycycline for 2 days or MCF-10a cells. Cells were treated with indicated concentrations of alisertib. Right panel: dose-response curve of MCF-10a cells treated with indicated concentrations of alisertib. Means and standard errors of 4 experimental replicates are depicted.

**Supplemental Figure 7:**

**(A)** BT-549-shBRCA2^dox^ were pretreated with doxycycline for 3 days, and treated with alisertib for an additional 2 days. Subsequently, genome-wide genomic sequencing of single cells was performed. Each line represents genomic reads mapped to genomic locations of a single cell. Ploidy state is color-coded as indicated.
