# Supplementary figures and images for: TPX2/Aurora kinase A signaling as a potential therapeutic target in genomically unstable cancer cells
Source: Oncogene. 2018 Sep 3;38(6):852–67. doi: 10.1038/s41388-018-0470-2 (PMC6367211; doi:10.1038/s41388-018-0470-2)

Supplemental Figure 1

A

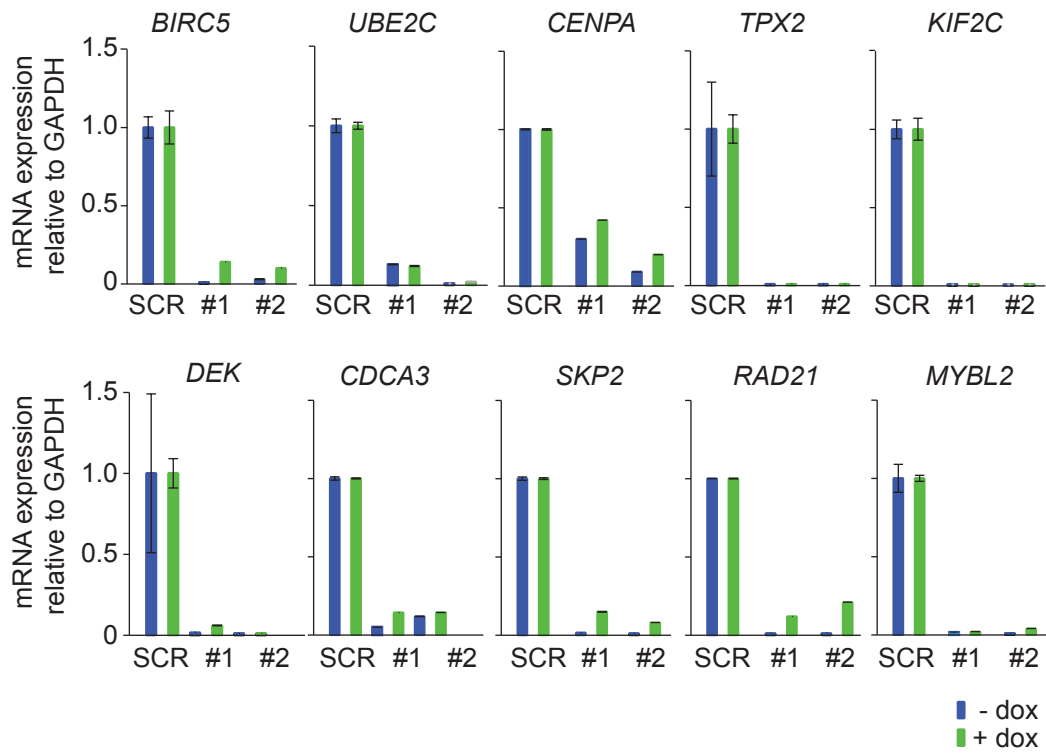

B

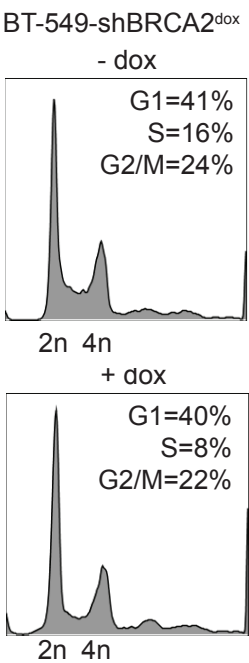

C

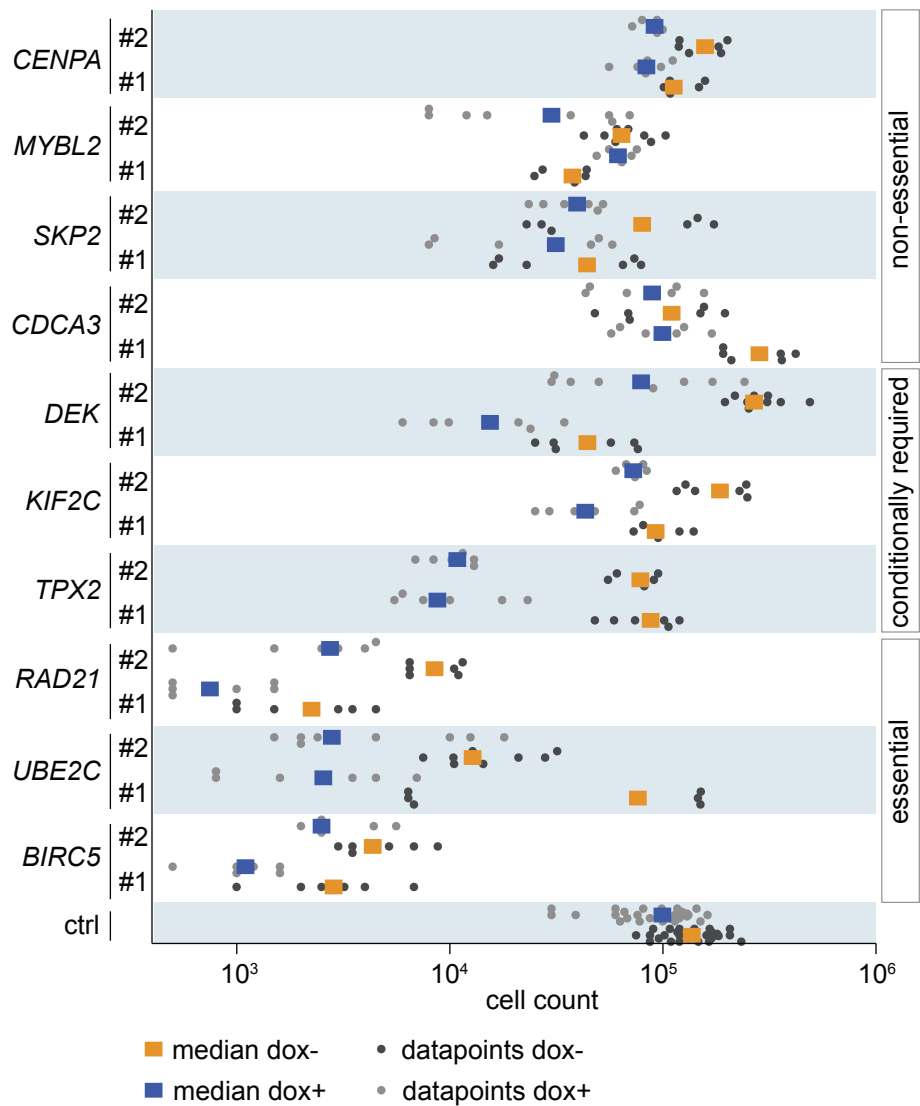

Supplement: Supplementary file 2 — Supplemental Figure 1 [file 41388_2018_470_MOESM2_ESM.pdf]

Supplemental Figure 2

A

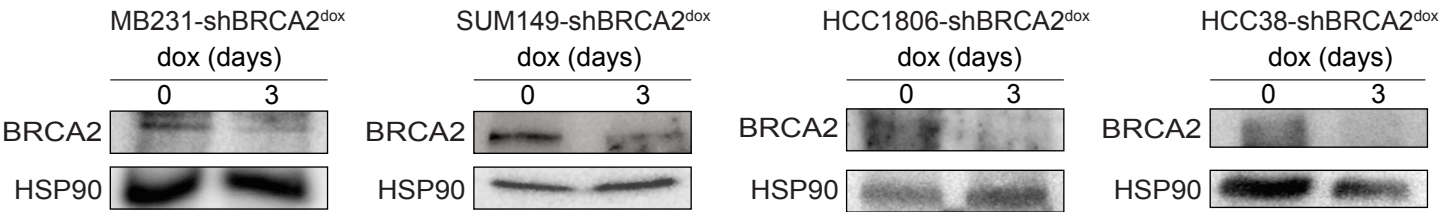

B

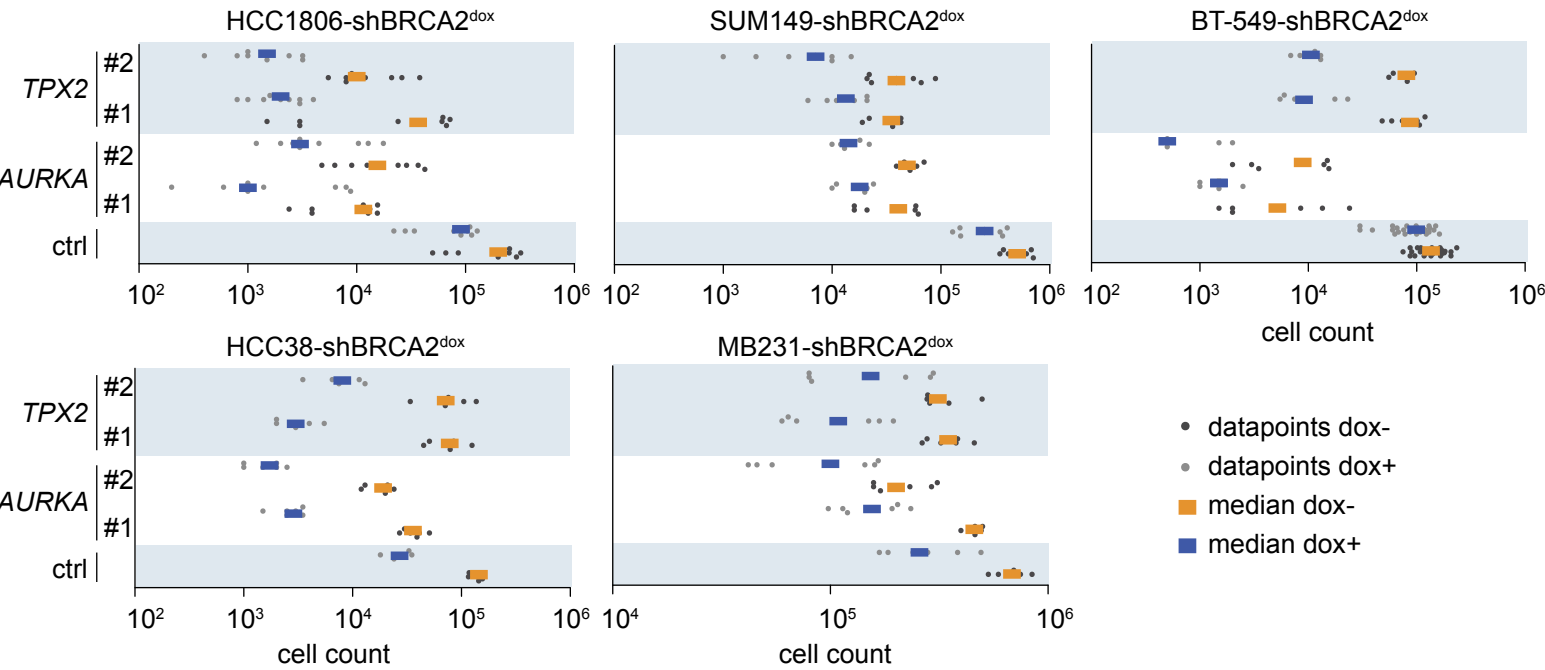

Supplement: Supplementary file 3 — Supplemental Figure 2 [file 41388_2018_470_MOESM3_ESM.pdf]

# Supplemental Figure 3

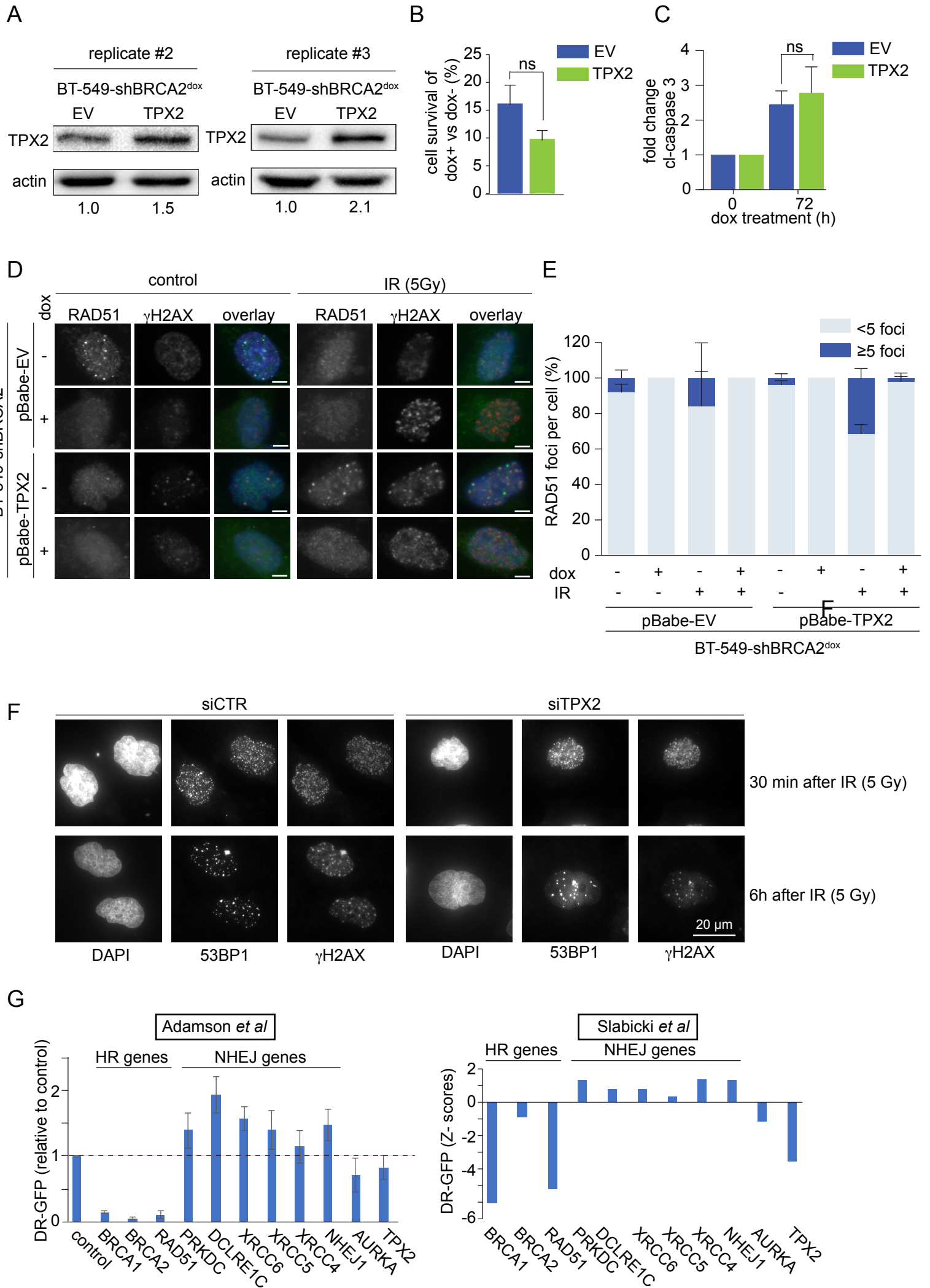

Supplement: Supplementary file 4 — Supplemental Figure 3 [file 41388_2018_470_MOESM4_ESM.pdf]

Supplemental Figure 4

A

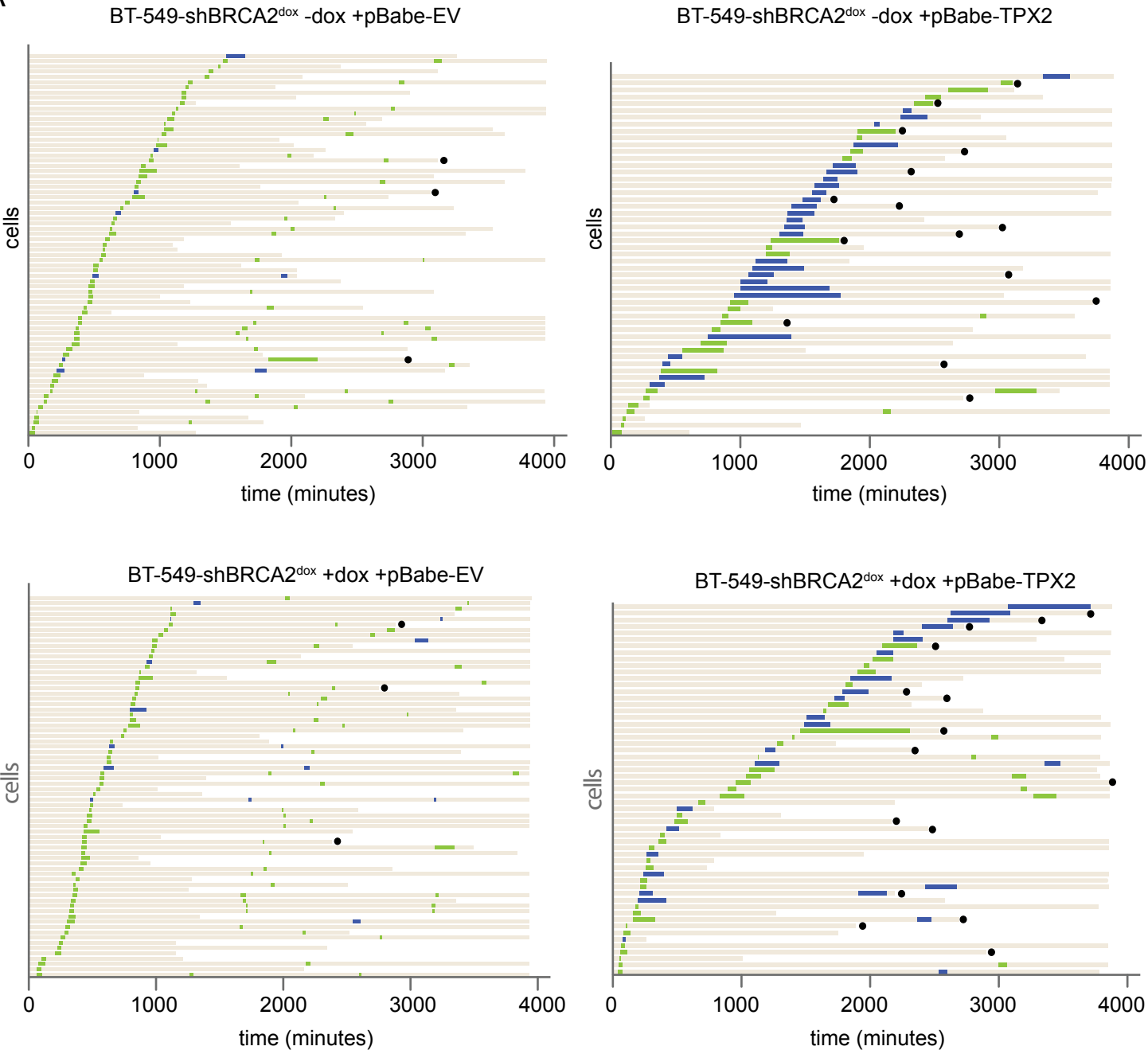

B

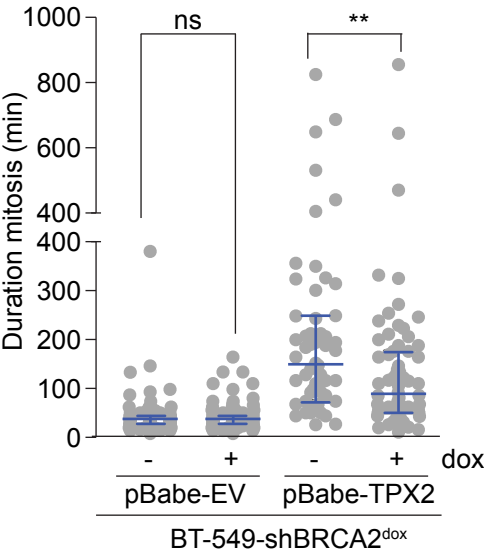

C

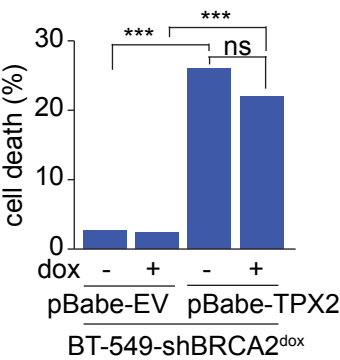

D

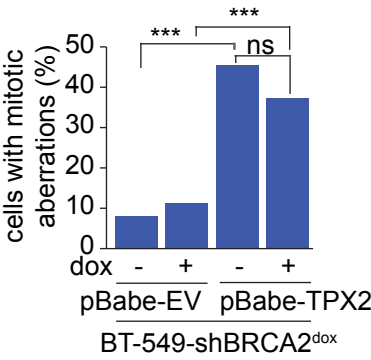

Supplement: Supplementary file 5 — Supplemental Figure 4 [file 41388_2018_470_MOESM5_ESM.pdf]

Supplemental Figure 5

A

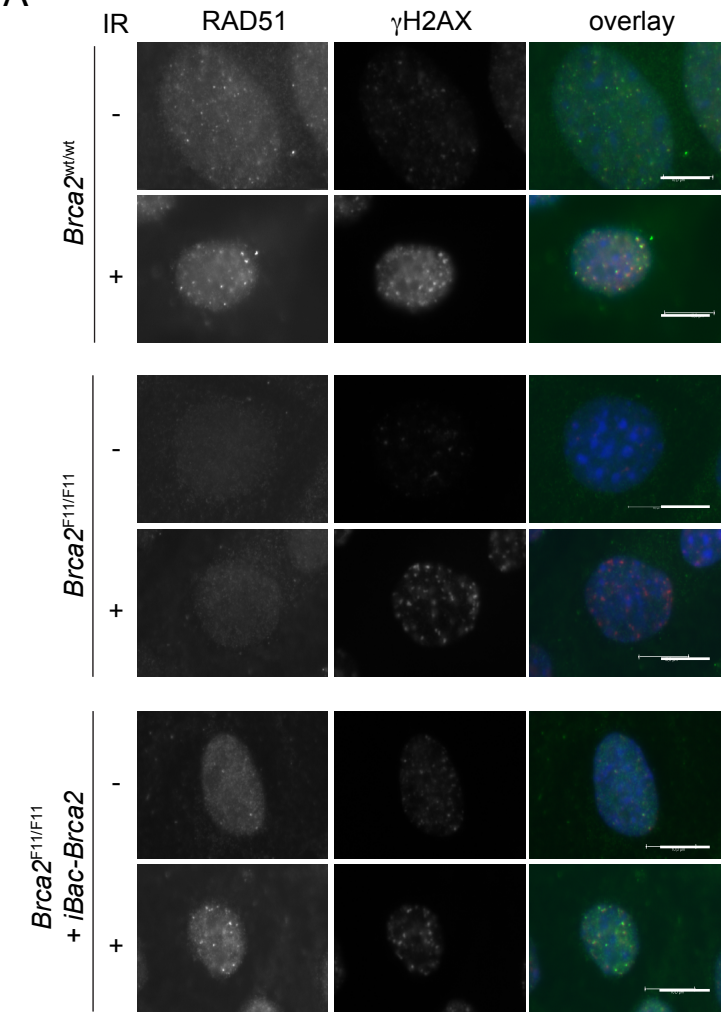

B

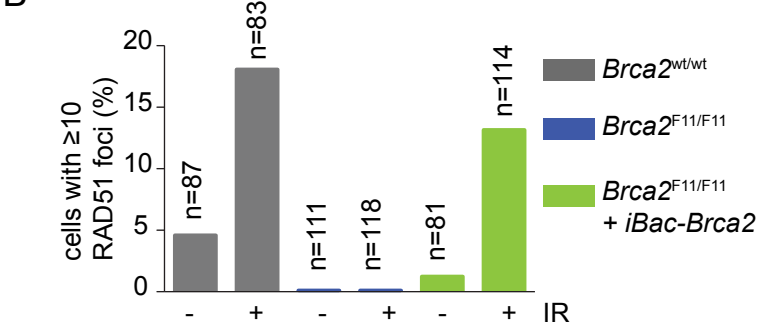

C

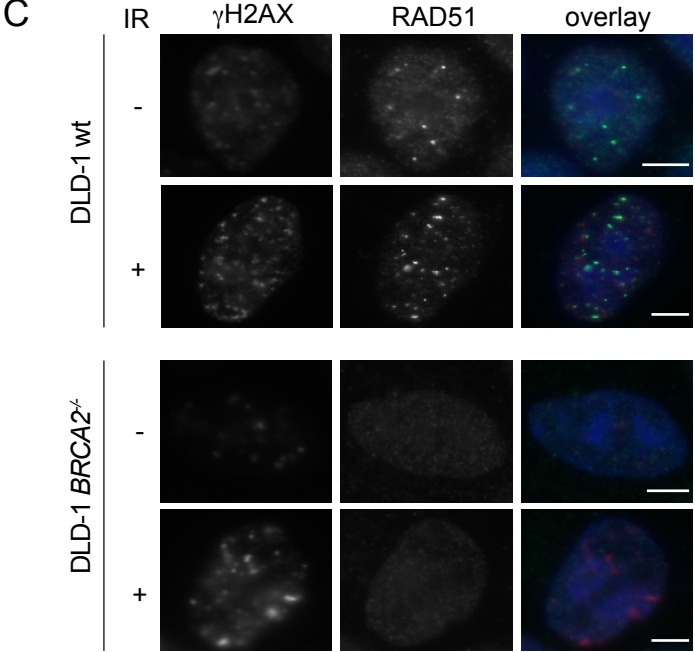

D

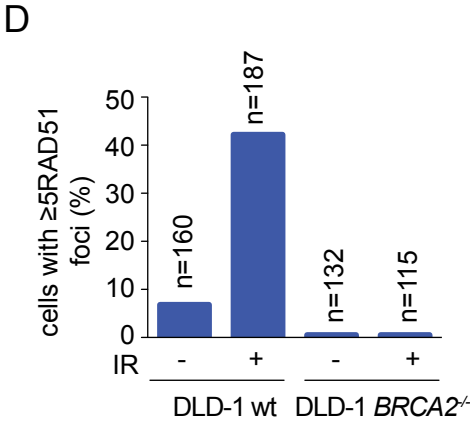

E

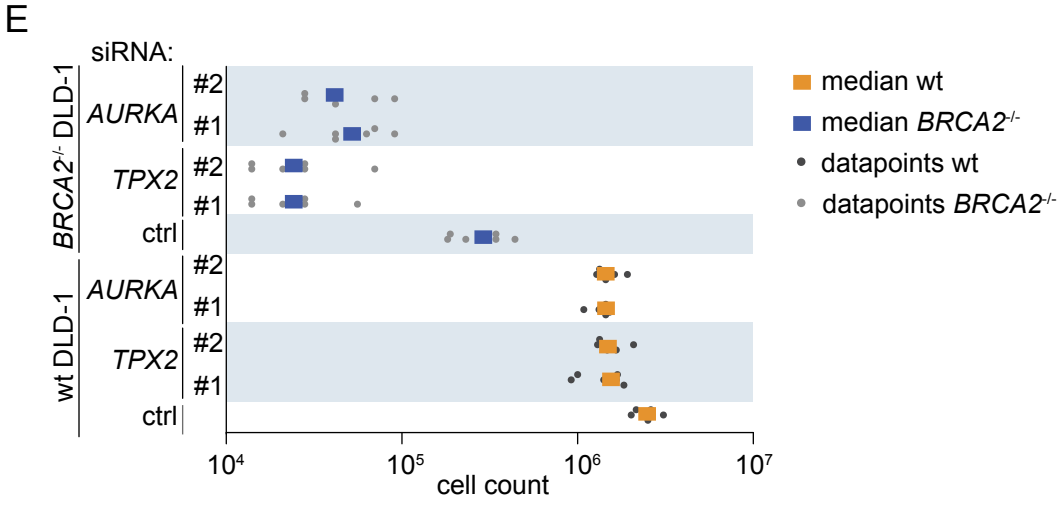

F

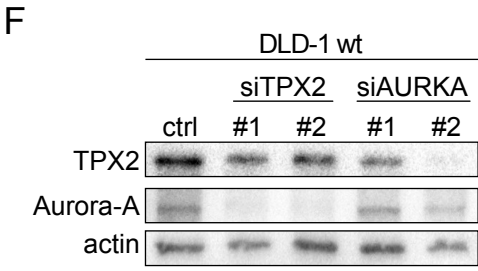

Supplement: Supplementary file 6 — Supplemental Figure 5 [file 41388_2018_470_MOESM6_ESM.pdf]

Supplemental Figure 6

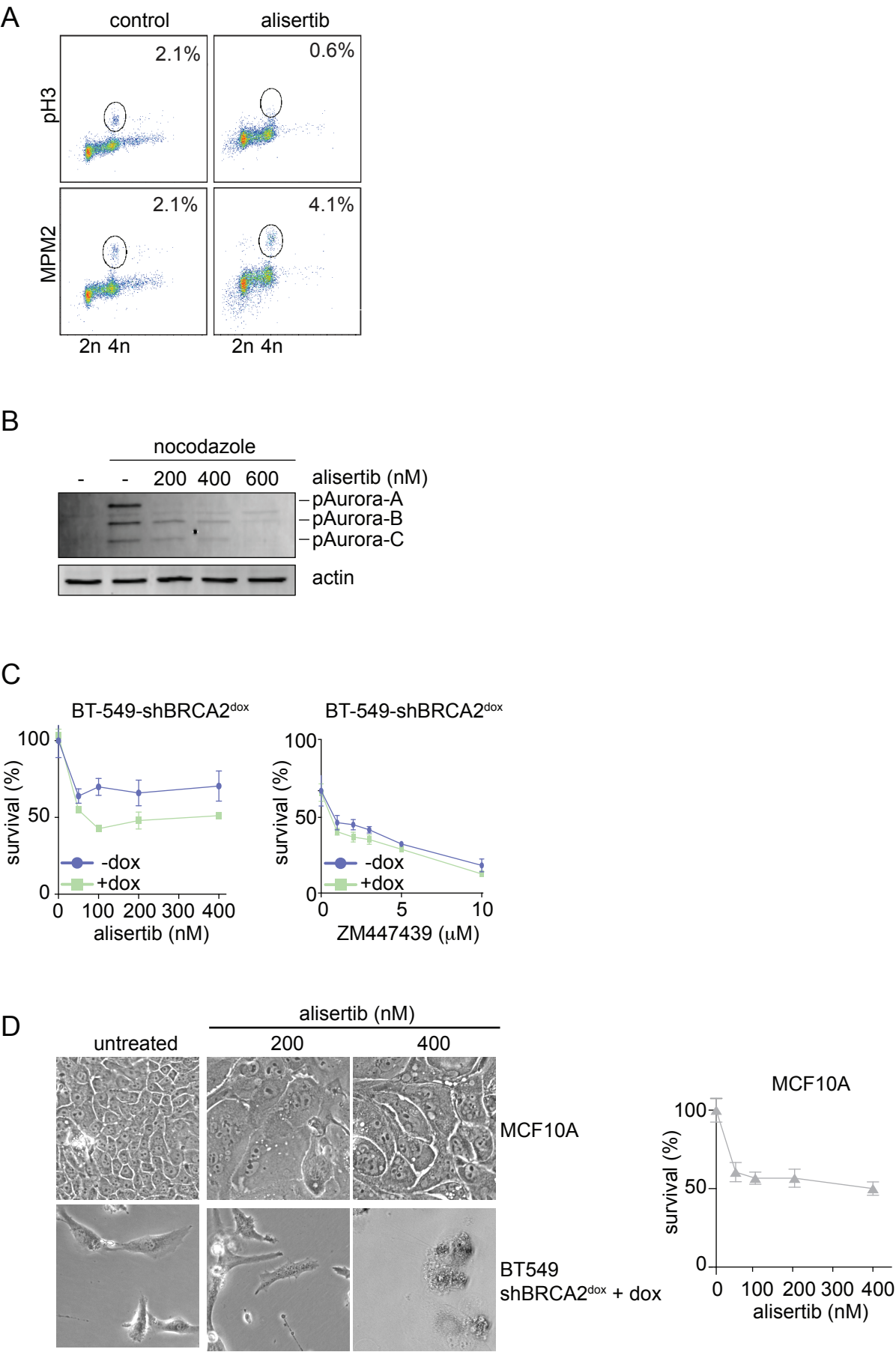

Supplement: Supplementary file 7 — Supplemental Figure 6 [file 41388_2018_470_MOESM7_ESM.pdf]

Supplemental Figure 7

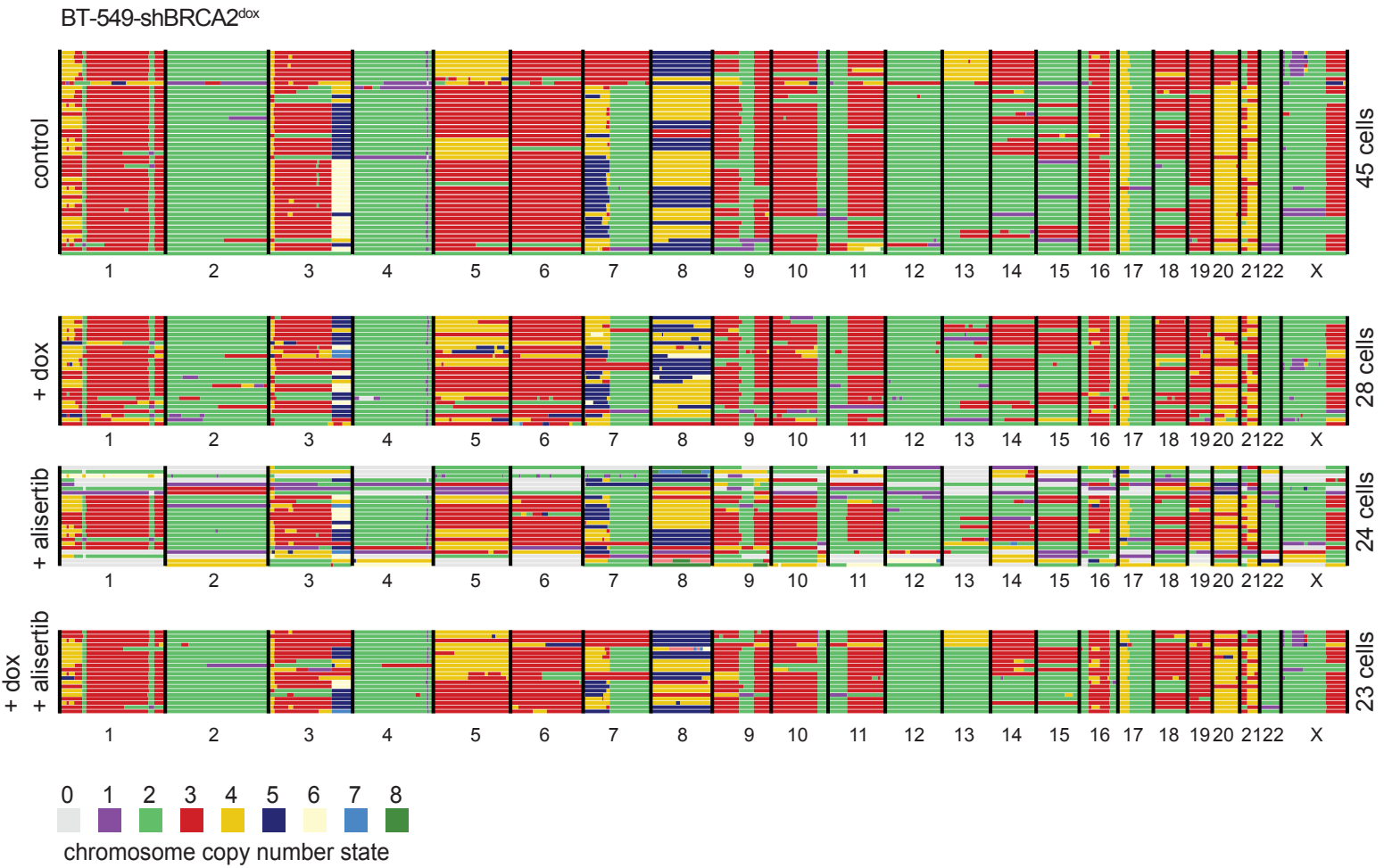

Supplement: Supplementary file 8 — Supplemental Figure 7 [file 41388_2018_470_MOESM8_ESM.pdf]
